# Comparative Biocompatibility and Odonto-/Osteogenesis Effects of Hydraulic Calcium Silicate-Based Cements in Simulated Direct and Indirect Approaches for Regenerative Endodontic Treatments: A Systematic Review

**DOI:** 10.3390/jfb14090446

**Published:** 2023-08-29

**Authors:** Amir-Ali Yousefi-Koma, Hadi Assadian, Sadra Mohaghegh, Hanieh Nokhbatolfoghahaei

**Affiliations:** 1 Dental Research Center, Research Institute of Dental Sciences, School of Dentistry, Shahid Beheshti University of Medical Sciences, Tehran 1983963113, Iran; 2 Department of Endodontics, Tehran University of Medical Sciences, Tehran 1417614418, Iran

**Keywords:** hydraulic calcium silicate-based cements, indirect pulp capping, direct pulp capping, MTA, Biodentine

## Abstract

Background: Regenerative dentistry is the operation of restoring dental, oral and maxillofacial tissues. Currently, there are no guidelines for the ideal cement/material in regenerative endodontic treatments (RET). Hydraulic calcium silicate-based cements (hCSCs) are currently the material of choice for RET. Objectives: This systematic review was conducted to gather all of the different direct and indirect approaches of using hCSCs in RET in vitro and in vivo, and to ascertain if there are any superiorities to indirect approaches. Methods and Materials: This systematic review was conducted according to the 2020 PRISMA guidelines. The study question according to the PICO format was as follows: Comparison of the biological behavior (O) of stem cells (P) exposed to hCSCs through direct and indirect methods (I) with untreated stem cells (C). An electronic search was executed in Scopus, Google Scholar, and PubMed. Results: A total of 78 studies were included. Studies were published between 2010 and 2022. Twenty-eight commercially available and eighteen modified hCSCs were used. Seven exposure methods (four direct and three indirect contacts) were assessed. ProRoot MTA and Biodentine were the most used hCSCs and had the most desirable results. hCSCs were either freshly mixed or set before application. Most studies allowed hCSCs to set in incubation for 24 h before application, which resulted in the most desirable biological outcomes. Freshly mixed hCSCs had the worst outcomes. Indirect methods had significantly better viability/proliferation and odonto-/osteogenesis outcomes. Conclusion: Biodentine and ProRoot MTA used in indirect exposure methods result in desirable biological outcomes.

## 1. Introduction

Regenerative dentistry is the operation of restoring and/or regenerating dental, oral and maxillofacial tissues and organs for therapeutic implementations [1,2,3,4]. Regenerative endodontic treatments (RET) are a large group of procedures assessed to maintain and regenerate dentine and pulpal tissues. Vital pulp therapy (VPT) sustains dental pulp vitality and maintains teeth [5]. Pulpotomy and direct pulp capping (DPC), induce the formation of regenerative dentine by human dental pulp stem cells (hDPSCs) in the treatment of exposed vital pulp [6]. Pulp capping materials develop a protective layer over the exposed vital pulp in pulpotomy, DPC, and indirect pulp capping (IPC) [7]. Ideal pulp capping materials must be biocompatible, have excellent sealing abilities, and promote migration, proliferation, and differentiation of hDPSCs [8,9]. Newly developed bioactive materials (e.g., bioactive glasses and calcium silicate-based cements) are produced/introduced every so often [10,11]. However, currently there are no guidelines for the ideal cement/material in RET.

Hydraulic calcium silicate-based cements (hCSCs) are inorganic restorative commercial cements, which are currently the material of choice for repair procedures and regeneration in RET (e.g., VPT, pulpotomy, DPC, IPC, apexogenesis, apexification, root-end filling, and perforation repair) [12,13,14,15]. hCSCs are bioactive, biocompatible, hold clinically acceptable sealing properties, and can induce the formation of regenerative hard tissues [7,16]. Tricalcium silicate (Ca_3_SiO_5_) and dicalcium silicate (Ca_2_SiO_4_) are two major components of hCSCs [17,18,19,20,21,22,23,24,25,26]. Before the introduction of mineral trioxide aggregate (MTA) in 1993 as the first commercially available hCSC, calcium hydroxide [Ca(OH)_2_] (CH) was commonly used as the main pulp capping material [27,28]. CH has marginal leakage, weak cohesive strength, and lacks adequate antibacterial effects [29]. Following the remarkable outcomes of utilizing MTA in different pulp capping and endodontic treatments, a variety of commercially available hCSCs have been introduced to clinicians (e.g., Biodentine (BD), TheraCal (TC), Emdogain (EG), Portland cement (PC), Bioaggregate (BA), calcium-enriched matrix (CEM), Endo sequence putty (ERRM), etc.) [30,31,32,33].

In endodontic treatments and DPC procedures, hDPSCs and other types of alveolodental stem cells are in direct contact with hCSCs [34]. hCSCs and their toxins in direct contact with stem cells are much more harmful to the stem cells compared to indirect contact [35,36]. Consequently, many studies have tested the outcome differences of hCSCs in direct and indirect contact with stem cells, to compare their proliferative and regenerative abilities in vitro [37,38,39,40,41]. When hCSCs are clinically applied for human patients, there is no precise way to evaluate their biological outcomes, except extraction of the teeth and laboratory analysis. Therefore, a lot of the reported outcomes regarding hCSCs used in clinics do not have enough evidence to prove the toxicity/biocompatibility of hCSCs in both direct and indirect contact. However, in vitro studies, if conducted according to global standards, can be a reliable simulation of the clinical interactions between stem cells and hCSCs. Clinicians can choose their kind of hCSC and the type of contact based on studies conducted in vitro that simulate clinical environments.

To the reviewers’ knowledge, there has been no comprehensive review executed on the comparison of viability/proliferation and the odonto-osteogenesis differentiation induction abilities of all of the commercially available hCSCs. Additionally, there is no review comparing the outcomes of different types of direct and indirect contacts in vitro. The main purpose of this systematic review was to gather all of the different direct and indirect approaches of using hCSCs in RET in vitro and in vivo, and to ascertain if there were any superiorities to indirect approaches when examined for biocompatibility and regeneration/differentiation abilities. Additionally, we sought to find the hCSCs with the most remarkable outcomes in each of the direct and indirect approaches in vitro, in order to help clinicians and scientists make an informed choice.

## 2. Results and Discussion

**Figure 1:**
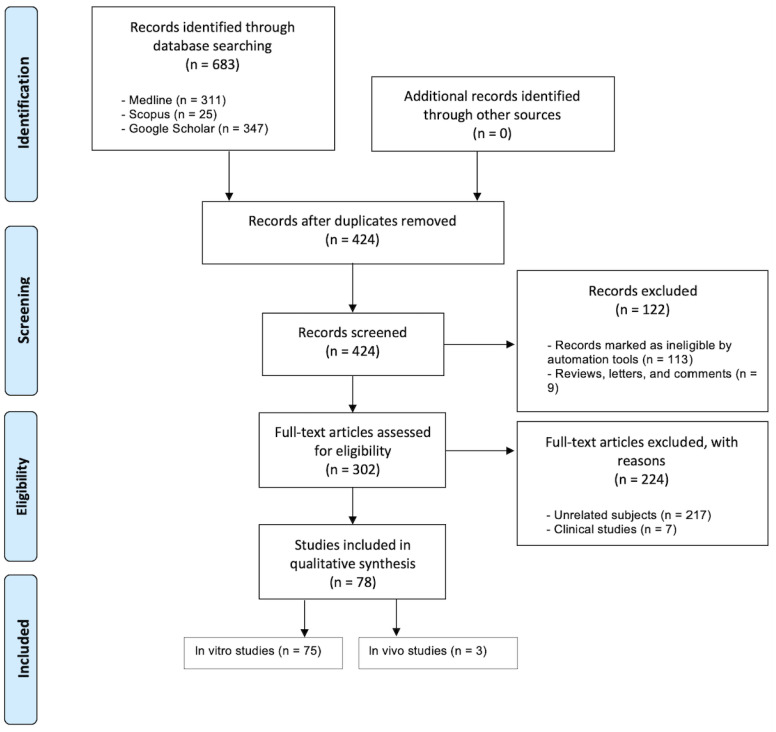
The PRISMA flow diagram of the identification and screening process.

The search queries and PRISMA flow diagram (according to the PRISMA 2020 guidelines [42]) of this systematic review are displayed in Table 1 and Figure 1, respectively.

### 2.1. Study Selection

**Figure 2:**
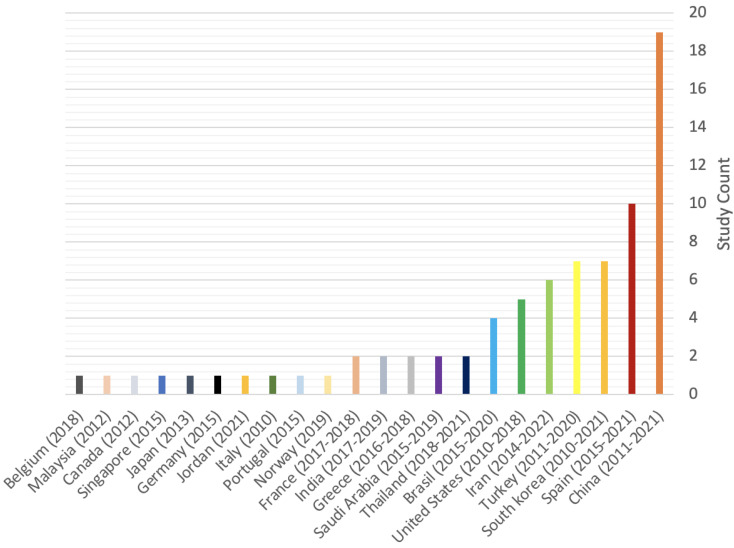
Distribution of included in vitro and in vivo studies.

Database screening was performed, a total of 683 articles were initially identified and 302 of them were assessed for eligibility (Figure 1). A total of 224 studies were excluded for the following reasons: clinical studies (n = 7) and unrelated subjects (n = 217). Hence, a total of 75 in vitro and 3 in vivo studies matched our inclusion criteria. Figure 2 showcases the distributions for all of the included studies and the range of years they were published in. All of the studies, their cells, cements, contact methods, and outcomes are detailed in Table 2 and Table 3 for in vitro and in vivo studies, respectively. All of the abbreviated forms used in this review are listed in Table S1.

### 2.2. Study Characteristics

#### 2.2.1. Types of hCSCs and Their Setting Times and Condition

**Figure 3:**
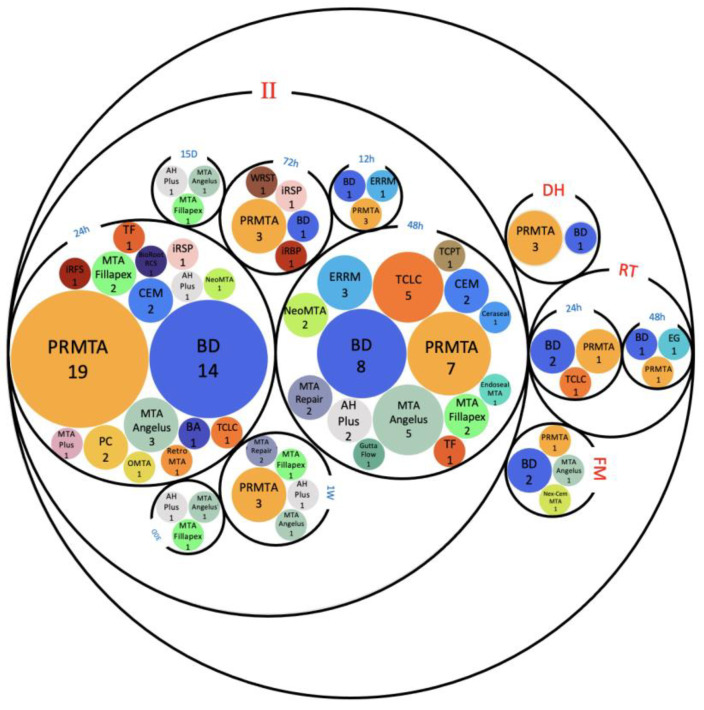
Setting times (12 h, 24 h, 48 h, 72 h, 1 week (1W), 15 days (15D) and 30 days (30D)) and setting conditions (i.e., in incubation **(II)**, dried heat **(DH)**, room temperature **(RT)**, and freshly mixed **(FM)**) of hCSCs. Abbreviations of included hCSCs: **BA:** Bioaggregate, **BD:** Biodentine, **EG:** Emdogain, **iRBP:** iRoot BP, **iRFS:** iRoot fast set, **iRSP:** iRoot SP, **OMTA:** OrthoMTA, **PC:** Portland cement, **PRMTA:** ProRoot MTA, **TCLC:** TheraCal LC, **TCPT:** TheraCal PT, **TF:** TotalFill, and **WRST:** well root ST.

A total of 46 hCSCs were used in our included studies: 28 commercially available hCSCs along with 18 different modifications of hCSCs. PRMTA (n = 40) and BD (n = 34) were the most frequently used cements amongst all of the studies [37,38,39,41,43,44,45,46,47,48,49,50,51,52,53,54,55,56,57,58,59,60,61,62,63,64,65,66,67,68,69,70,71,72,73,74,75,76,77,78,79,80,81,82,83,84,85,86,87,88,89,90,91,92,93,94], followed by TCLC (n = 9), MTA Angelus (n = 8), MTA Fillapex (n = 6), ERRM (n = 6), CEM (n = 5), PC (n =4), AH Plus (n = 4), Neo MTA (n = 3), MTA Repair (n = 3), iRFS (n = 3), iRSP (n = 2), TF (n = 2) and NeoPutty (n =2) (Tables S2 and S3). The rest of the hCSCs were used only in one study. The setting times and conditions in which cements were left prior to applying them to cells are mentioned in Figure 3.

#### 2.2.2. Types of Cells

In total, 12 different types of stem cells were examined in the included studies: (1) human dental pulp stem cells (hDPSCs) (n = 48) [37,40,41,43,45,46,47,49,52,53,54,57,59,60,62,63,64,65,66,67,68,69,73,74,79,82,83,84,85,87,89,90,91,92,93,94,95,96,97,98,99,100,101,102,103,104,105], (2) stem cells from apical papilla (SCAP) (n = 8) [50,71,72,76,79,82,105,106], (3) human bone marrow stem cells (hBMSCs) (n = 8) [46,49,59,66,70,78,84,105], (4) human periodontal ligament stem cells (hPDLSCs) (n = 7) [49,68,88,98,106,107,108], (5) stem cells from human exfoliated deciduous teeth (SHED) (n = 4) [38,39,55,109], (6) human tooth germ stem cells (hTGSCs) (n = 3) [49,110,111], (7) rat bone marrow stem cells (rBMSCs) (n = 2) [44,76], (8) human umbilical vein endothelial cells (hUVESCs) (n = 1) [51], (9) rat dental pulp stem cells (rDPSCs) (n = 1) [56], (10) raw 264.7 cells (n = 1) [46], (11) C3H10T ½ cells (n = 1) [77], and (12) C2C12 cells (n = 1) [58].

**Table 2:** All 75 of the included in vitro studies, their tested and control groups, interventions, methods of assessment, evaluation periods and results.

Author/Year	Cements and Materials (Setting Times and Conditions) (Dilutions/Concentrations)	Cells/Interventions	Methods of Assessment	Results		
				Attachment (A)/ Migration (M)	Viability/ Proliferation	Odonto-/ Osteogenesis
Youssef et al. [43]/2019	1. PRMTA (48 h set RT) 2. CH (48 h set RT) 3. EG 4. BD (48 h set RT) 5. NC	hDPSCs/ Direct1	1. Attachment, viability and proliferation: MTT (D 3) 2. Odonto-/Osteogenesis: RT-PCR (D 7 and 14) 3. Migration: NM	- A: NM - M: NM	D3: NC > EG >> PRMTA > CH > BD	1. DSPP: 1.1. D7: EG >> CH > BD > PRMTA > NC 1.2. D14: CH >> BD > NC > EG > PRMTA 2.ALP: 2.1. D7: CH >> NC > EG > BD > PRMTA 2.2. D14: BD >> CH > PRMTA > NC > EG 3.OPN: 3.1. D7: CH >> PRMTA > EG > BD > NC 3.2. D14: BD >> CH > NC > PRMTA > EG
Sun et al. [103]/2020	1. NeoPutty 2.E RRM 3. NC 4. IRM (CP)	hDPSCs and hPDLSCs/ Indirect1	1. Attachment, viability and proliferation: MTT (D 3) 2. Odonto-/Osteogenesis: NM 3. Migration: NM	- A: NM - M: NM	D3: NC > NeoPutty > ERRM	NM
Lu et al. [44]/2019	1. iRBP (72 h set II and dried for 24 h) (0.02, 0.2, 1.0 and 2.0 mg/mL) 2. PRMTA (CP) (72 h set II and dried for 24 h) (2 mg/mL) 3. NC	rBMMSCs/ Indirect3	1. Attachment, viability and proliferation: CCK-8 (D 0, 1, 3, 5 and 7) 2. Odonto-/Osteogenesis: ALP (D 3, 5 and 7), Western blot (0, 15, 30 and 60 min), RT-PCR (D 0, 3 and 7) and ARS (D14) 3. Migration: NM	- A: NM - M: NM	At all-time points: BP iRBP (0.2 mg/mL) ≈ NC	1.ALP activity: D3, D5 and D7: iRBP 0.2 mg/mL >> iRBP 0.02 mg/mL > NC > iRBP 2 mg/mL > iRBP 1 mg/mL 2.DSPP, OSX, OPN and ALP: 1.1. D0: NSD 1.2. D3 and D7: iRBP (0.2 mg/mL) >> NC 3. RUNX2: 2.1. D0: NC >> iRBP (0.2 mg/mL) 2.2. D3 and D7: iRBP (0.2 mg/mL) >> NC 4.ARS: iRBP >> NC
Tu et al. [46]/2020	1. PRMTA (24 h set II) 2. CAMTA: ProRoot MTA with TAF (24 h set II) 3. NC	hDPSCs and Raw 264.7 cells/Direct1	1. Attachment, viability and proliferation: Prestoblue and ELISA both at 12 h, D1 and D2 2. Odonto-/Osteogenesis: ARS (D 7 and 14) and ELISA (D 1 and 2) 3. Migration: NM	- A: 12 h, D1 and D2: CAMTA > PRMTA ≈ NC - M: NM	12 h, D1 and D2: CAMTA > PRMTA ≈ NC	1. DSPP and ALP: D7 and D14: CAMTA >> PRMTA > NC 2. ARS: D7 and D14: CAMTA >> PRMTA
Pedano et al. [47]/2018	1. Exp. cement: containing PPL (FM) (10%, 25%, 50% and 100% concentrations) 2. Nex-Cem MTA (FM) (10%, 25% and 50% concentrations) 3. BD (FM) (10%, 25% and 50% concentrations) 4. ZnOE (CP) (FM) (10%, 25% and 50% concentrations) 5. NC	hDPSCs/ Indirect1	1. Attachment, viability and proliferation: XTT (D 1, 4 and 7) 2. Odonto-/Osteogenesis: RT-PCR (D 4, 10 and 14) 3. Migration: WHA (D1)	- A: NM - M: 1. 10% concentration: the peak for each experiment; NexMTA > NC > Exp. > BD 2. NexMTA: 10%, 25% and 50% concentrations: NexMTA ≈ NC 3. Exp.: 10% and 25% concentrations: Exp. ≈ NC 4. BD: NC >> BD	1. 10% concentration: 1.1. D1: Exp. > BD = NexMTA = NC > ZnOE 1.2. D4: NC > Exp. > BD > NexMTA > ZnOE 1.3. D7: BD > NC > Exp. > NexMTA > ZnOE 2. 25% concentration: 2.1. D1: NexMTA > Exp. > NC > BD > ZnOE 2.2. D4: NC > Exp. > BD > NexMTA > ZnOE 2.3. D7: NC > BD > Exp. > NexMTA > ZnOE 3. 50% concentration: 3.1. D1: NexMTA > Exp. > NC > BD > ZnOE 3.2. D4: NC > Exp. > NexMTA > BD > ZnOE 3.3. D7: NC > BD > Exp. > NexMTA > ZnOE	1.DSPP: 1.1. D4: NC >> BD > Exp. > NexMTA 1.2. D10: BD >> NexMTA > NC > Exp. 1.3. D14: BD >> Exp. > NC > NexMTA 2.ALP: 2.1. D4: NC >> NexMTA > Exp. = BD 2.2. D10: BD > Exp. > NexMTA = NC 2.3. D14: Exp. >> NC > NexMTA > BD 3.OCN: 3.1. D4: NC > Exp. > BD > NexMTA 3.2. D10: BD >> NexMTA > NC > Exp. 3.3. D14: BD ≈ Exp. >> NexMTA > NC
Ali et al. [48]/2019	1. PRMTA (24 h set II) (1:2, 1:4,1:8 and 1:16 dilutions) 2. BD (24 h set II) (1:2, 1:4,1:8 and 1:16 dilutions) 3. TF (24 h set II) (1:2, 1:4,1:8 and 1:16 dilutions) 4. NC (1:2, 1:4,1:8 and 1:16 dilutions)	hBMSCs/ Indirect1	1. Attachment, viability and proliferation: MTT (D 1,3 and 7) 2. Odonto-/Osteogenesis: RT-PCR (6 h and D 1,3 and 7) and ELISA (D 1,3 and 7) 3. Migration: NM	- A: NM - M: NM	1. D1, D3 and D7: 1:8 and 1:16 dilutions: NSD 2. D3 and D7: 1:2 and 1:4 dilutions: NC >> PRMTA ≈ BD ≈ TF	1. ALP: 1.1. D7: TF = NC > PRMTA > BD 1.2. 6 h: BD >> NC ≈ PRMTA ≈ TF 2. COL1A: D7: PRMTA ≈ BD >> TF >> NC 3. OC: D1: BD >> NC ≈ PRMTA ≈ TF D7: NSD
Couto et al. [104]/2020	1. White MTA 2. CH 3. COP 4. MTA + COP 5. CH + COP 6. Cells in mineralizing medium (CP) 7. NC	hDPSCs/ Indirect3	1. Attachment, viability and proliferation: MTT (D 1, 2 and 3) 2. Odonto-/Osteogenesis: RT-PCR (D21) and ARS (D21) 3. Migration: WHA (12 h, D1 and D2)	- A: NM - M: 1.CH: no migration was observed 2. 12 h, D1 and D2: CH + COP > MTA + COP > NC > COP >> MTA 3. D1 and D2: CH + COP >> MTA + COP ≈ NC ≈ COP ≈ MTA	D3: NC > COP > CH + COP > MTA > MTA + COP >> CH	1. DSPP and OCN: MTA + COP >> CH + COP ≈ NC ≈ COP ≈ MTA 2. ARS: COP >> CH + COP > MTA > MTA + COP > CH > CP > NC
Olcay et al. [49]/2019	1. PRMTA (72 h set II) 2. BD (72 h set II) 3. WRST (72 h set II) 4. Dycal (72 h set II) 5. NC	hDPSCs, hPDLSCs and hTGSCs/ Indirect2	1. Attachment, viability and proliferation: MTS (D 1, 3, 7, 10 and 14) 2. Odonto-/Osteogenesis: NM 3. Migration: NM	- A: NM - M: NM	1. hDPSCs: D14: PRMTA > BD > WRST > NC >> Dycal 2. hTGSCs: D14: NC > WRST = BD > PRMTA >> Dycal 3. hPDLSCs: D7: PRMTA >> BD ≈ Dycal ≈ NC ≈ WRST	NM
Güven et al. [110]/2013	1. MTA Fillapex (24 h set II) 2. iRSP (24 h set II) 3. AH Plus Jet (24 h set II) 4. NC	hTGSCs/ Indirect2	1. Attachment, viability and proliferation: MTS and SEM both at D 1, 3, 7 and 14 2. Odonto-/Osteogenesis: NM 3. Migration: NM	- A: iRSP ≈ NC - M: NM	1. D1: NC > AH > iRSP >> Fillapex 2. D3: NC > iRSP > AH >> Fillapex 3. D7: NC >> AH > iRSP >> Fillapex 4. D14: iRSP > NC > AH >> Fillapex	NM
Schneider et al. [50]/2014	1. PRMTA: with plain α-MEM (1 h set RT (FM) or 24 h set II): 2. PRMTA: with calcium-enriched media (3.0, 0.3 and 0.03 mmol dilutions of CaCl_2_) (1 h set RT (FM) and 24h set II): 3. PRMTA: with 2% FBS (1 h set RT (FM) and 24 h set II): 4. FBS (0%,2% and 10%) and CaCl_2_ media (NC)	SCAP/ Indirect2	1. Attachment, viability and proliferation: WST-1 (D 1, 3, 5, 7, 9, 11 and 14) 2. Odonto-/Osteogenesis: NM 3. Migration: TMA (0.5, 1, 3, 6, 12, 24, 48 and 72 h)	- A: NM - M: 1. 0.5 h to 6 h: Significantly higher in 24 h set PRMTA with plain α-MEM 2. 24, 48 and 72 h: significantly higher in PRMTA + CaCl_2_ 3. FBS 2% and 10%: significantly induced early and short-term migration	1. PRMTA with 0.3 and 0.03 mmol CaCl_2_ media: significant increase from D1 to D7 and decreased afterwards 2. PRMTA with 2% FBS: significantly lower than NC at D7 onwards	NM
Bortoluzzi et al. [37]/2015	1. MTA Angelus (FM or 24 h set II) 2. BD (FM or 24 h set II) 3. TCLC 4. IRM (CP) 5. NC	hDPSCs/ Indirect1 and Indirect2	1. Attachment, viability and proliferation: XTT (D3 for Indirect2 and D4 for Indirect1) and Flow cytometry (D3) 2. Odonto-/Osteogenesis: RT-PCR (D7) 3. Migration: NM	- A: NM - M: NM	1. At the end of the fourth aging cycle: NC ≈ MTA ≈ BD >> TCLC 2. FM: all cements were cytotoxic	1. DSPP and DMP1, ALP and BSP: BD ≈ MTA >> TCLC > NC 2. OCN, and Runx2: BD ≈ MTA >> NC > TCLC
Jun et al. [105]/2019	1. Ceria-incorporated MTA (CMTA: 2% and 4%) 2. NC 3. MTA (CP)	hDPSCs/ Indirect2	1. Attachment, viability and proliferation: MTS (D1) 2. Odonto-/Osteogenesis: RT-PCR (D7), ARS (D21) and ALP (D 7 and 14) 3. Migration: NM	- A: NM - M: NM	D1: CMTA >> MTA ≈ NC	ARS and ALP activity: CMTA ≈ MTA >> NC
Costa et al. [51]/2015	1.PRMTA (24 h set II) (1:2, 1:5, 1:10, and 1:20 dilutions) 2.MTA Plus (24 h set II) (1:2, 1:5, 1:10, and 1:20 dilutions) 3.MTA Fillapex (24 h set II) (1:2, 1:5, 1:10, and 1:20 dilutions) 4.BD (24 h set II) (1:2, 1:5, 1:10, and 1:20 dilutions) 5.NC	hBMSCs and hUVECs/ Indirect1	1.Attachment, viability and proliferation: Cell lysates (D 1, 7, 14 and 21) 2.Odonto-/Osteogenesis: ALP (D 7, 14, 21) 3.Migration: NM	- A: NM - M: NM	1. D21: PRMTA (1:20) ≈ MTA Plus (1:20) >> Fillapex ≈ BD ≈ NC 2. At all time points: PRMTA (1:20) ≈ MTA Plus (1:20) ≈ NC >> MTA Fillapex ≈ BD (1:2)	ALP activity: D21: PRMTA (1:20) ≈ MTA Plus (1:20) >> control >> Fillapex ≈ BD (1:2 and 1:5)
D’Antò et al. [52]/2010	1. PRMTA (24 h set II) 2. PC (24 h set II) 3. NC 4. FBS 20% (CP)	hDPSCs/ Direct1	1. Attachment, viability and proliferation: Alamar blue (D 1, 3, 5, 7, 14, 21 and 28) and CLSM (D1) 2. Odonto-/Osteogenesis: NM 3. Migration: TMA (18 h)	- A: D1: PRMTA > PC ≈ NC - M: CP >> PRMTA >> PC ≈ NC	D14, D21 and D28: PRMTA >> PC ≈ NC	NM
Collado-González et al. [53]/2017	1. MTA Angelus (48 h set II) (1:1, 1:2 and 1:4 dilutions) 2. BD (48 h set II) (1:1, 1:2 and 1:4 dilutions) 3. TCLC (48 h set II) (1:1, 1:2 and 1:4 dilutions) 4. IRM (48 h set II) (CP) (1:1, 1:2 and 1:4 dilutions) 5. NC	hDPSCs/ Indirect1	1. Attachment, viability and proliferation: MTT (D 1,2 and 3) and SEM (D3) 2. Odonto-/Osteogenesis: NM 3. Migration: WHA (D 1 and 2)	- A: MTA ≈ BD ≈ NC >> TCLC ≈ IRM - M: 1.D1: 1.1. 1:1 dilution: NC ≈ BD >> MTA > IRM > TCLC 1.2. 1:2 dilution: TCLC >> BD > MTA > TCLC > IRM 1.3. 1:4 dilution: NC >> BD > TCLC > IRM > MTA 2.D2: 2.1. 1:1 dilution: NC = BD >> MTA > TCLC > IRM 2.2. 1:2 and 1:4 dilutions: NC ≈ BD >> MTA > IRM > TCLC	1. 1:1 and 1:2 dilution at D3: BD >> NC >> IRM ≈ TCLC 2. 1:4 dilution at D3: BD >> NC ≈ TCLC ≈ IRM	NM
Agrafioti et al. [54]/2016	1. PRMTA (1 h or 24 h set RT) 2. BD (24 h set RT) 3. NC	hDPSCs/ Direct2	1. Attachment, viability and proliferation: MTT (D 4 and 7) 2. Odonto-/Osteogenesis: NM 3. Migration: NM	- A: NM - M: NM	1. D4: BD >> PRMTA (24 h set) ≈ NC >> PRMTA (1 h set) 2. D7: PRMTA (24 h set) ≈ BD >> NC >> PRMTA (1 h set)	NM
Hasweh et al. [55]/2021	1. BD (15 min DH) (4 concentrations: 20 mg/mL, 2 mg/mL, 0.2 mg/mL and 0.02 mg/mL) 2. NC	SHED/ Indirect3	1. Attachment, viability and proliferation: MTT (D 1, 2, 3, 4, 5 and 6) and CAA (D1) 2. Odonto-/Osteogenesis: NM 3. Migration: WHA (D1) and TMA (D1)	- A: 0.2 mg/mL BD ≈ 0.02 mg/mL BD ≈ 2 mg/mL BD > NC - M: 0.2 mg/mL BD ≈ 0.02 mg/mL BD ≈ NC >> 2 mg/mL BD	0.2 mg/mL BD ≈ 0.02 mg/mL BD > 2 mg/mL BD > NC >> 20 mg/mL BD	NM
Wang et al. [56]/2014	1. PRMTA (24 h DH) (0.002, 0.02, 0.2, 2, and 20 mg/mL concentrations) 2. NC	rDPSCs/ Indirect3	1. Attachment, viability and proliferation: MTT (D 1, 3, 5 and 7) and FCM 2. Odonto-/Osteogenesis: RT-PCR (D 3 and 7), Western blot (minutes 0, 15, 30 and 60), ARS and ALP (D 3 and 5) 3. Migration: NM	- A: NM - M: NM	0.2 mg/mL PRMTA ≈ NC >> 2 mg/mL PRMTA > 20 mg/mL PRMTA	In 0.2 mg/mL MTA: 1. DSPP: PRMTA D7 > PRMTA D3 >> NC 2. ALP and OCN: PRMTA D7 > PRMTA D3 > NC 3. Runx2 and OSX: PRMTA D3 >> NC ≈ PRMTA D7 4. ARS and ALP activity: 0.2 mg/mL PRMTA >> NC
Widbiller et al. [57]/2015	1. PRMTA (24 h set II) 2. BD (24 h set II) 3. GIC (24 h set II) 4. Human dentin disks 5. NC	hDPSCs/ Direct1	1. Attachment, viability and proliferation: MTT (D 1, 3, 5 7, 10 and 14) and SEM (D1) 2. Odonto-/Osteogenesis: qRT-PCR (D 7, 14 and 21) and ALP (D 3, 7 and 14) 3. Migration: NM	- A: D1: Cell spreading and attachment was observed in BD - M: NM	1. D14: BD ≈ PRMTA >> NC ≈ dentin disks 2. GIC: significantly cytotoxic	1. ALP activity: dentin disks > BD >> PRMTA > NC 2. DSPP: D14: PRMTA >> BD ≈ dentin disks ≈ NC D21: BD >> PRMTA ≈ dentin disks ≈ NC 3. ALP: D3 and D14: dentin disks >> PRMTA ≈ NC >> BD 4. Runx2: NC >> PRMTA >> BD 5. COL1A1: D7: PRMTA ≈ BD >> NC
Athanasiadou et al. [38]/2018	1. BD (24 h set II) (1:1, 1:2, 1:4, 1:8, 1:16, 1:32, 1:64 and 1:128 dilutions) 2. NC	SHED/Direct1 (staining) and Indirect1 (MTT)	1. Attachment, viability and proliferation: SEM (D3), MTT (D 1, 3 and 5) and LDA (D3) 2. Odonto-/Osteogenesis: RT-PCR (D 7 and 14), ARS (D14) 3. Migration: NM	- A: BD: Adhesion and spreading were observed - M: NM	D3: BD >> NC	1. DSPP, ALP, Runx2 and BMP2: BD >> NC 2. ARS: NSD
Wang et al. [112]/2017	1. MTA (0.002, 0.02, 0.2, 2, 20 mg/mL concentrations) 2. Mineralization-inducing medium (MM) 3. MTA (2 mg/mL) + MM 4. Mouse IgG isotype antibodies (NC) 5. Gapdh (CP)	hPDLSCs/ Indirect3	1. Attachment, viability and proliferation: CCK-8 (D 1, 3, 5, 7 and 9) 2. Odonto-/Osteogenesis: RT-PCR (D 3 and 7), Western blot (0, 15, 30 and 60 min), ARS (D14) and ALP (D 3 and 5) 3. Migration: NM	- A: NM - M: NM	2 mg/mL MTA: NSD	1. RUNX2, OCN, OSX, COL-I, OPN, DMP1, ALP, and DSP: MTA >> NC 2. ARS and ALP activity: MTA + MM > MM > MTA >> NC
Matsumoto et al. [58]/2013	1.PRMTA (24 h set II) 2.NC	C2C12/ Indirect2	1.Attachment, viability and proliferation: CCK-8 (D 1, 3, 5 and 7) 2.Odonto-/Osteogenesis: RT-PCR (D 1, 3, 5 and 7) 3.Migration: NM	- A: NM - M: NM	1. D7: PRMTA >> NC 2.D3: NSD	Runx2: PRMTA >> NC
Ajlan et al. [113]/2015	1. MTA (0.02, 0.2 and 2.0 mg/mL concentrations) 2. EMD (0.05, 0.1 and 0.2 mg/mL concentrations) 3. PDGF (0.000005, 0.00001and 0.00002 mg/mL concentrations) 4. NC 5. Cells in osteoinduction medium (OT) (reference control)	hDPSCs/ Indirect3	1. Attachment, viability and proliferation: - 2. Odonto-/Osteogenesis: ALP (D14) and ARS (D14) 3. Migration: NM	- A: NM - M: NM	NM	1.ALP activity: 1.1. Lowest concentrations: MTA > EMD > PDGF 1.2. Middle concentrations: EMD >> PDGF > MTA 1.3. Highest concentrations: EMD >> PDGF >> MTA 2.ARS: EMD > MTA >> OT > PDGF > NC
Paranjpe et al. [59]/2010	1. PRMTA (48 h set II) 2. NC 3. BMP-4 (CP) 4. NAC (CP)	hDPSCs/ Direct1	1. Attachment, viability and proliferation: Flow cytometry (D1) 2. Odonto-/Osteogenesis: RT-PCR (D 1, 4 and 7) 3. Migration: NM	- A: NM - M: NM	D1: NSD	1. Runx2: 1.1. D1 and D4: BMP-4 >> PRMTA > NC 1.2. D7: almost non-existent in all groups 2.DSPP: 2.1. D1: BMP-4 > PRMTA > NC 2.2. D4 and D7: PRMTA >> BMP-4 > NC 3.OCN: 3.1. D1: BMP-4 > PRMTA = NC 3.2. D4: BMP-4 >> PRMTA > NC 3.3. D7: PRMTA >> BMP-4 > NC 4. ALP: 4.1. D1 and D7: BMP-4 >> PRMTA > NC 4.2. D4: BMP-4 > PRMTA > NC
Araújo et al. [60]/2017	1. PRMTA 2. BD 3. CH 4. NC 5. Culture medium with 20% FBS (CP)	hDPSCs/ Indirect3	1. Attachment, viability and proliferation: MTT (D 1, 3, 5 and 7) and SRB (D 1, 3, 5 and 7) 2. Odonto-/Osteogenesis: RT-PCR (D 1, 7, 14 and 21) 3.Migration: Cell Tracker^TM^ Green CMFDA (D1)	- A: NM - M: BD > PRMTA >> CH > CP > NC	1. MTT: 1.1. D1 and D3: NSD 1.2. D5 and D7: BD >> PRMTA ≈ CH >> NC 2. SRB: NSD	DMP1: 1. D1: NSD 2. D7: PRMTA > CH >> NC >> BD 3. D14: PRMTA > CH > BD >> NC 4. D21: PRMTA > BD > CH >> NC
Tsai et al. [39]/2018	1. PRMTA (1 week set II) 2. NC	SHEDs/Direct1 and Indirect2	1. Attachment, viability and proliferation: WST-1 (D 1, 2 and 3) 2. Odonto-/Osteogenesis: NM 3. Migration: NM	- A: NC >> PRMTA - M: NM	1. Direct: NC >> PRMTA 2. Indirect: D1 and D3: NC >> PRMTA D2: NSD	NM
Vanka et al. [61]/2019	1. PRMTA (24 h set II) 2. PRP (5% and 10% concentrations) 3.P RMTA combined with PRP 4. NC	hBMSCs/ Direct1	1. Attachment, viability and proliferation: MTT (D 3, 7 and 14) and CAA (D3) 2. Odonto-/Osteogenesis: ARS (D14) 3. Migration: NM	- A: PRMTA + 10%PRP >> PRMTA + 5%PRP > PRMTA > 10%PRP > 5% PRP > NC - M: NM	1. D3, D7 and D14: NSD 2. D7 and D14: MTA ≈ PRMTA/5% PRP ≈ PRMTA/10%PRP >> NC	ARS: PRMTA + PRP 10% >> PRMTA + 5%PRP > 10%PRP > PRMTA = 5% PRP > NC
Kulan et al. [62]/2018	1. PRMTA with additives: (24 h set II) 1.1. Distilled water (DW) 1.2. Na_2_HPO_4_ 2.5% 1.3. CaCl_2_ 5% 2. PRMTA (24 h set II) (CP) 3. NC	hDPSCs/ Direct1	1. Attachment, viability and proliferation: MTS (D 1, 7 and 21) 2. Odonto-/Osteogenesis: RT-PCR (D 14 and 21) and ALP (D 7 and 14) 3. Migration: NM	- A: NM - M: NM	1. D1: NSD 2. D7: NC > PRMTA + DW = PRMTA + CaCl_2_ > PRMTA + Na_2_HPO_4_ 3. D21: NC >> PRMTA + CaCl_2_ > PRMTA + DW > PRMTA + Na_2_HPO_4_	ALP activity: D7 and D14: PRMTA + CaCl_2_ >> PRMTA + Na_2_HPO_4_> NC > PRMTA + DW
Lee et al. [63]/2010	1. PRMTA (24 h set II) 2. Calcium phosphate cements (CPCs) 3. CPC-Ch (CPC with chitosan) 3. PC 4. NC	hDPSCs/ Direct1	1. Attachment, viability and proliferation: MTS (D 1, 7 and 14) and SEM (D7) 2. Odonto-/Osteogenesis: RT-PCR (D 1, 7 and 14) and ALP (D 1, 7 and 14) 3. Migration: NM	- A: NSD - M: NM	1. D1 and D7: NC >> PC > CPC-Ch > CPC = PRMTA 2. D14: NC >> PC = PRMTA > CPC-Ch > CPC	1. DSPP: 1.1. D1: PRMTA > CPC = CPC-Ch > PC > NC 1.2. D7: CPC > CPC-Ch > PRMTA > PC >> NC 1.3. D14: CPC-Ch > CPC > PC > PRMTA >> NC 2. DMP1: 2.1. D1: CPC-Ch > CPC > PC > PRMTA >> NC 2.2. D7: PRMTA > PC = CPC-Ch > CPC >> NC 2.3. D14: PRMTA > PC >> CPC > CPC-Ch > NC 3. ALP activity: D1, D7 and D14: PC > CPC-Ch > CPC > PRMTA >> NC 4. BSP: 4.1. D1: PRMTA > PC > CPC-Ch > CPC > NC 4.2. D7: PC > CPC > CPC-Ch >> PRMTA >> NC 4.3. D14: PC > CPC-Ch > CPC > PRMTA > NC 5. OPN: 5.1. D1: CPC-Ch > CPC > PRMTA > PC >> NC 5.2. D7: CPC-Ch > CPC >> PRMTA > PC > NC 5.3. D14: CPC > PC > CPC-Ch > PRMTA > NC 6. ON: 6.1. D1: PRMTA > CPC > CPC-Ch >> PC > NC 6.2. D7: CPC > CPC-Ch > PRMTA >> PC > NC 6.3. D14: PC = CPC > CPC-Ch > PRMTA > NC
Tomás-Catalá et al. [64]/2017	1. BD (48 h set II) (1:1, 1:2 and 1:4 dilutions) 2. NeoMTA Plus (48 h set II) (1:1, 1:2 and 1:4 dilutions) 3. MTA repair HP (48 h set II) (1:1, 1:2 and 1:4 dilutions) 4. NC	hDPSCs/ Indirect1	1. Attachment, viability and proliferation: MTT (D 1, 2 and 3) and SEM (D3) 2. Odonto-/Osteogenesis: NM 3. Migration: WHA (D 1 and 2)	- A: BD >> HP ≈ NeoMTA ≈ NC - M: 1. D1: 1.1. 1:1 dilution: BD > NeoMTA > HP > NC 1.2. 1:2 dilution: NeoMTA > BD > HP = NC 1.3. 1:4 dilution: BD > NeoMTA = NC > HP 2.D2: 2.1. 1:1 dilution: BD > NC >> NeoMTA > HP 2.2. 1:2 dilution: BD > NC > NeoMTA >> HP 2.3. 1:4 dilution: BD > NC > NeoMTA > HP	1. D1: 1:1. 1:2 and 1:4 dilutions: NSD 2. D2: 2.1. 1:1 dilution: BD > HP > NeoMTA > NC 2.2. 1:2 dilution: BD > HP = NC > NeoMTA 2.3. 1:4 dilution: BD >> HP = NC > NeoMTA 3. D3: 3.1. 1:1 dilution: BD >> NeoMTA > HP > NC 3.2. 1:2 dilution: BD >> NeoMTA = HP = NC 3.3. 1:4 dilution: BD >> NC = NeoMTA > HP	NM
Guven et al. [114]/2011	1. MTA 2. Dycal 3. EMD 4. MTA + EMD 5. Dycal coated with EMD 6. NC 7. Regular tissue culture plate (TCP)	hTGSCs/ Direct1	1. Attachment, viability and proliferation: MTT (D2) and SEM (D14) 2. Odonto-/Osteogenesis: RT-PCR (D14) and ALP (D24) 3. Migration: NM	- A: EMD ≈ TCP >> MTA ≈ MTA + EMD ≈ NC >> Dycal - M: NM	1. D2: EMD > MTA > Dycal + EMD > MTA + EMD > Dycal > NC 2. EMD coated Dycal: EMD coating significantly reduced Dycal’s cytotoxicity	1. DSPP: EMD > MTA > NC 2. ALP activity: NC > EMD > TCP > MTA
Sun et al. [65]/2019	1. BD (set II) 2. iRFS (set II) 3.NC	hDPSCs/ Direct1	1. Attachment, viability and proliferation: SEM (D2) and LDA (D 1, 3 and 7) 2. Odonto-/Osteogenesis: RT-PCR (D 1, 3 and 7) 3. Migration: TMA (D7)	- A: NSD - M: D7: iRFS >> BD > NC	1. D1 and D3: iRFS > BD = NC 2. D7: iRFS >> BD = NC	1. ALP: 1.1. D1 and D7: NC >> iRFS > BD 1.2. D3: NC >> BD > iRFS 2. COL1: 2.1. D1: iRFS >> > BD 2.2. D3: iRFS > NC = BD 2.3. D7: BD > iRFS > NC 3. OCN: 3.1. D1: iRFS >> BD > NC 3.2. D3: NC >> BD > iRFS 3.3. D7: NC > iRFS > BD
Niu et al. [36]/2015	1. PRMTA (24 h set II) 2. Quick-set2 (experimental CS cement with oxide) (24 h set II) 3. IRM (CP) 4. NC	hDPSCs/ Indirect2	1. Attachment, viability and proliferation: MTT (D1), flow cytometry (D3) and CyQUANT (D3) 2. Odonto-/Osteogenesis: NM 3. Migration: NM	- A: NM - M: NM	1. First cycle: Quick-set2 was significantly cytotoxic 2. Third cycle: NSD	NM
Zhao et al. [66]/2011	1. PRMTA (1 week set II) (20, 10, 2, 1, 0.2, 0.1, 0.02, and 0.002 mg/mL concentrations) 2. NC	hDPSCs/ Indirect1	1. Attachment, viability and proliferation: MTT (D 1, 3 and 5) 2. Odonto-/Osteogenesis: RT-PCR (6 h, 12 h, D1 and D2) 3. Migration: NM	- A: NM - M: NM	1. In 10 and 20 mg/mL: cytotoxic at all time points 2. D1, D3 and D5: 2 mg/mL PRMTA = 1 mg/mL PRMTA > 0.2 mg/mL PRMTA > 0.1 mg/mL PRMTA > 0.02 mg/mL PRMTA > 0.002 mg/mL PRMTA > NC	1. DSPP: PRMTA (0.2 mg/mL) D2 > D1 > 12 h > 6 h >> NC 2. BSP: PRMTA (0.2 mg/mL) 12 h > D1 > D2 >> 6 h > NC 3. OCN: PRMTA (0.2 mg/mL) D2 > D1 > 12 h >> 6 h > NC 4. COL1 and ALP: PRMTA (0.2 mg/mL) 12 h > D1 > D2 > 6 h >> NC
Yu et al. [67]/2016	1. Experimental cement: containing resin monomer (MAE-DB) and Portland cement (PC) 2. PRMTA (48 h set II) 3. MAE-DB 4. PC 5. NC 6. Cells cultured with osteogenic medium (CP)	hDPSCs/ Indirect1	1. Attachment, viability and proliferation: CCK-8 (D 1, 2 and 3) and CAA (1 h) 2. Odonto-/Osteogenesis: RT-PCR (D14), ARS (D14) and ALP (D 3, 5, 7 and 9) 3. Migration: WHA (D1) and TMA (D1)	- A: 1 h: PRMTA = PC > Exp. >> NC - M: 1.TMA: PRMTA >> PC > Exp. > NC 2.WHA: MTA ≈ PC > NC > Exp.	1. D1: NC >> PRMTA = PC > Exp. > MAE-DB 2. D2: PRMTA = PC >> NC > Exp. >> MAE-DB 3. D3: NC = PRMTA = PC = Exp. >> MAE-DB	1. ALP activity: 1.1. D3: NSD 1.2. D5: PRMTA > PC = Exp. >> CP > NC 1.3. D7 and D9: PRMTA = PC > Exp. >> CP > NC 2. ARS: PRMTA = PC >> Exp. > CP > NC 3. DSPP: PC > PRMTA > Exp. >> CP > NC 4. OCN and BMP1: PRMTA > PC > Exp. >> CP > NC 5. ON: PRMTA = PC > Exp. >> CP > NC 6. ALP: PC >> PRMTA > Exp. > CP > NC
Tomás-Catalá et al. [40]/2017	1. NeoMTA Plus (48 h set II) (1:1, 1:2 and 1:4 dilutions) 2. MTA Angelus (48 h set II) (1:1, 1:2 and 1:4 dilutions) 3. MTA Repair HP (48h set II) (1:1, 1:2 and 1:4 dilutions) 4. NC	hDPSCs/ Direct1 and Indirect1	1. Attachment, viability and proliferation: MTT (D 1, 2 and 3) and SEM (D3) 2. Odonto-/Osteogenesis: NM 3. Migration: WHA (D 1 and 2)	- A: NSD - M: 1.D1: NeoMTA ≈ NC >> HP ≈ Angelus 2.D2: NC > HP >> Angelus ≈ NeoMTA	1. D1: NSD 2. D2: 2.1. 1:1 dilution: Angelus > HP >> NeoMTA > NC 2.2. 1:2 dilution: Angelus > HP > NeoMTA > NC 2.3. 1:4 dilution: Angelus >> HP > NC >> NeoMTA 3. D3: 3.1. 1:1 dilution: Angelus > HP > NeoMTA >> NC 3.2. 1:2 dilution: NSD 3.3. 1:4 dilution: NC = NeoMTA > Angelus >> HP	NM
Chen et al. [68]/2016	1. Newly developed bioceramic cement (RRM) (72 h set II) 2. PRMTA (72 h set II) 3. NC	hDPSCs, hBMSCs and hPDLSCs/ Direct1	1. Attachment, viability and proliferation: MTT (D 1, 3 and 5) and SEM (D3) 2. Odonto-/Osteogenesis: SEM (D3) 3. Migration: NM	- A: D3: NSD - M: NM	1. hDPSCs: 1.1. D1: PRMTA > RRM > NC 1.2. D3: RRM > PRMTA > NC 1.3. D5: RRM >> PRMTA >> NC 2. hBMSCs: 2.1. D1: NC > PRMTA > RRM 2.2. D3: PRMTA > RRM > NC 2.3. D5: PRMTA > RRM >> NC 3. hPDLSCs: 3.1. D1: RRM > NC > PRMTA 3.2. D3: PRMTA = RRM >> NC 3.3. D5: RRM >> PRMTA >> NC	NM
Asgary et al. [69]/2014	1. PRMTA (24 h set II) 2. CEM (24 h set II) 3. Gapdh (CP) 4. Growth medium (GM) (NC) 5. Differentiation medium (DM) (NC)	hDPSCs/ Direct1	1. Attachment, viability and proliferation: SEM (D 1, 3, 7 and 14) 2. Odonto-/Osteogenesis: RT-PCR (D 1, 3, 7 and 14) and ARS (D14) 3. Migration: NM	- A: NM - M: NM	NSD	1. ARS: PRMTA >> NC 2. DSPP: 2.1. D1: NSD 2.2. D3 and D7: PRMTA > CEM > DM >> GM 2.3. D14: PRMTA > DM > CEM >> GM 3. DMP1: 3.1. D1: PRMTA = CEM = DM > GM 3.2. D3: PRMTA > DM > CEM >> GM 3.3. D7: PRMTA > CEM > DM >> GM 3.4. D14: CEM > DM > PRMTA >> GM 4. ALP: 4.1. D1: NSD 4.2. D3, D7 and D14: DM >> PRMTA = CEM >> GM
Peters et al. [70]/2016	1. PRMTA (24 h set II) 2. BD (24 h set II) 3. NC 4. Cells with cobalt chloride (CP)	SCAP/ Direct1	1. Attachment, viability and proliferation: XTT (D 1, 3 and 7) and PCM (D 1 and 3) 2. Odonto-/Osteogenesis: NM 3. Migration: NM	- A: D1 and D3: NSD - M: NM	1. D1: PRMTA > BD >> NC > CP 2. D3: PRMTA > NC > BD > CP 3. D7: PRMTA > BD > NC = CP	NM
Wongwatanasanti et al. [71]/2018	1. PRMTA (24 h set II) 2. RetroMTA (24 h set II) 3. BD (24 h set II) 4.NC 5. Odonto-/osteogenic induction medium (CP)	SCAP/ Indirect2	1. Attachment, viability and proliferation: MTT (D 1, 3, 7 and 14) 2. Odonto-/Osteogenesis: ARS (D 7, 14 and 21) 3. Migration: NM	- A: NM - M: NM	1. D1: NSD 2. D3 and D7: BD > RetroMTA > PRMTA > NC 3.D14: RetroMTA = PRMTA > BD >> NC	ARS: BD ≈ CP >> PRMTA ≈ RetroMTA ≈ NC
Seo et al. [115]/2013	1. MTA 2. NC	hDPSCs/ Indirect2	1. Attachment, viability and proliferation: NM 2. Odonto-/Osteogenesis: RT-PCR (D14) 3. Migration: RT-PCR (D14)	- A: NM - M: NSD	NM	1. DSPP: NSD 2. DMP1: MTA >> control
Sultana et al. [72]/2017	1. PRMTA (48 h set: 2 h to 3 h RT, and the rest II) 2. BD (48 h set: 2 h to 3 h RT, and the rest II) 3. ERRM (48 h set: 2 h to 3 h RT, and the rest II) 4. GIC (48 h set: 2 h to 3 h RT, and the rest II) 5. NC	hBMSCs/ Direct1	1. Attachment, viability and proliferation: MTT (D 1, 3, 5 and 7) and LDA (D 7 and 21) 2.O donto-/Osteogenesis: ALP (D21) 3. Migration: -	- A: D7 and D21: GIC >> BD ≈ ERRM >> NC - M: NM	1. D1: NSD 2. D3: BD >> PRMTA = ERRM = NC > GIC 2. D5: NC >> ERRM > PRMTA > BD > GIC 3. D7: NC >> ERRM > PRMTA > BD > GIC	ALP activity: ERRM ≈ PRMTA >> GIC ≈ NC >> BD
Luo et al. [73]/2014	1. BD (4 concentrations: 0.02, 0.2, 2.0 and 20.0 mg/mL) 2. NC	hDPSCs/ Indirect3	1. Attachment, viability and proliferation: MTT (D 1, 3, 5 and 7) and BrdU (D1) 2. Odonto-/Osteogenesis: NM 3. Migration: WHA (D1) and TMA (D1)	- A: D1: 0.2 mg/mL BD >> 2 mg/mL BD > NC = 0.02 mg/mL BD > 20 mg/mL BD - M: WHA and TMA: 0.2 mg/mL BD >> NC	1. D1: 0.02 mg/mL BD > 0.2 mg/mL BD > 2 mg/mL BD > NC > 20 mg/mL BD 2. D3, D5 and D7: 0.2 mg/mL BD >> 2 mg/mL BD > 0.02 mg/mL BD = NC > 20 mg/mL BD	NM
Luo et al. [74]/2014	1. BD (0.2 and 2.0 mg/mL concentrations) 2. Cells cultured in mineralization medium (CP) 3. NC	hDPSCs/ Indirect3	1. Attachment, viability and proliferation:- 2. Odonto-/Osteogenesis: ALP (D 1, 3, 7, 10 and 14) and qRT-PCR (D14) 3. Migration: NM	- A: NM - M: NM	NM	1. ALP activity: 1.1. D1: NSD 1.2. D3, D7, D10 and D14: 0.2 mg/mL BD >> 2 mg/mL BD > CP > NC 2. DSPP, DMP1, OCN and BSP: 0.2 mg/mL BD >> NC
Yan et al. [75]/2014	1. PRMTA (24 h set DH) (0.0002, 0.002, 0.02, 0.2, 2.0 and 20 mg/mL concentrations) 2. Histone H1 and beta*-actin (internal controls) 3.NC	SCAP/ Indirect3	1. Attachment, viability and proliferation: IF (0, 0.25, 0.5, 1, and 3 h) and WB (D 1, 3, 5, 7 and 9) 2. Odonto-/Osteogenesis: RT-PCR (D 3 and 7), ALP (D 3 and 5) 3. Migration: NM	- A: NM - M: NM	At any time point: NSD	1. ALP activity: 2 mg/mL PRMTA >> 20 mg/mL PRMTA > 0.2 mg/mL PRMTA > 0.02 mg/mL PRMTA > 0.002 mg/mL PRMTA > NC 2. DSPP, ALP, Runx2 and OCN: PRMTA >> NC
Wang et al. [76]/2013	1.PRMTA (24 h set DH) (0.002, 0.02, 0.2, 2.0 and 20 mg/mL concentrations) 2. NC 3. Gapdh (internal control)	rBMSCs/ Indirect3	1. Attachment, viability and proliferation: MTT (D 1, 3, 5 and 7) 2. Odonto-/Osteogenesis: ALP (D 3 and 5), ARS (D14), RT-PCR (D 3 and 7) and WB (D 3 and 7; each day at 0, 30, 60 and 90 min) 3. Migration: NM	- A: NM - M: NM	D3 and D5: 0.02 mg/mL PRMTA > NC > 0.002 mg/mL PRMTA > 0.2 mg/mL = 2 mg/mL PRMTA >> 20 mg/mL PRMTA	1. ALP activity: 2.1. D3: 0.02 mg/mL PRMTA > 0.002 mg/mL PRMTA > NC > 0.2 mg/mL = 2 mg/mL PRMTA >> 20 mg/mL PRMTA 2.2. D5: 0.02 mg/mL PRMTA > 0.002 mg/mL PRMTA > NC > 0.2 mg/mL PRMTA > 2 mg/mL PRMTA >> 20 mg/mL PRMTA 2. DSPP, ALP, Runx2, OCN and OSX: PRMTA >> NC
Du et al. [108]/2020	1. MTA (0.02, 0.2, 2.0, 10 and 20 mg/mL concentrations) 2. NC	SCAP/ Indirect3	1. Attachment, viability and proliferation: CCK-8 (D 1, 3, 5 and 7) 2. Odonto-/Osteogenesis: ALP (D 3 and 5), RT-PCR (D5) and WB (0, 5, 15, 30, 60 and 120 min) 3.Migration: NM	- A: NM - M: NM	1. D1: 2 mg/mL MTA > 0.2 mg/mL MTA > 0.02 mg/mL MTA > NC >> 10 mg/mL MTA > 20 mg/mL MTA 2. D3 and D7: 0.2 mg/mL MTA > 0.02 mg/mL MTA > 2 mg/mL MTA > NC >> 10 mg/mL MTA > 20 mg/mL MTA 3. D5: NC > 0.02 mg/mL = 0.2 mg/mL = 2 mg/mL MTA > 10 mg/mL MTA > 20 mg/mL MTA	1. ALP activity: D3 and D5: 0.2 mg/mL MTA >> 2 mg/mL MTA > 0.02 mg/mL MTA > NC 2. DSPP and OCN: 0.2 mg/mL MTA >> 2 mg/mL MTA > 0.02 mg/mL MTA > NC 3. Runx2 and BSP: 0.2 mg/mL MTA > 2 mg/mL MTA >> 0.02 mg/mL MTA > NC
Lee et al. [77]/2014	1. MTA (24 h set II) (1:1, 1:2, 1:4, 1:10 and 1:50 dilutions) 2. BA (24 h set II) (1:1, 1:2, 1:4, 1:10 and 1:50 dilutions) 3. BD (24 h set II) (1:1, 1:2, 1:4, 1:10 and 1:50 dilutions) 4. NC	C3H10T1/2 cells/ Indirect1	1. Attachment, viability and proliferation: XTT (D5) 2. Odonto-/Osteogenesis: ALP (D 5 and 7) and RT-PCR (D 1, 2 and 3) 3. Migration: NM	- A: NM - M: NM	1. In 1:1, 1:2 and 1:4 dilutions: BA ≈ MTA >> BD 2. In 1:10 and 1:50 dilutions: NSD	1.ALP activity: 1.1. D5: BD > BA > MTA > NC 1.2. D7: MTA > BD > BA > NC 2. ALP: 2.1. D1 and D2: MTA >> BA > NC > BD 2.2. D3: BA > MTA >> NC > BD 3. OC: 3.1. D1: BA > MTA >> NC > BD 3.2. D2 and D3: MTA > BA >> NC > BD 4. BSP: 4.1. D1: BD >> NC > BA > MTA 4.2. D2: MTA >> BA > NC > BD 4.3. D3: BA >> MTA > BD > NC
Miller et al. [78]/2018	1. BD (12 h set II) 2. ERRM (12 h set II) 3. ERRM-FS (12 h set II) 4. PRMTA (12 h set II) 5. NC	SCAP/ Direct1	1. Attachment, viability and proliferation: OZBlue (D7) 2. Odonto-/Osteogenesis: ARS (D21) and RT-PCR (D21) 3. Migration: NM	- A: NM - M: NM	D7: ERRM >> BD > ERRM-FS = NC >> PRMTA	1. DSPP: ERRM >> BD > ERRM-FS > PRMTA > NC 2. ALP: ERRM >> BD > PRMTA = NC > ERRM-FS 3. Runx2: PRMTA = BD = ERRM = ERRM-FS = NC 4. IBSP: PRMTA >> BD > ERRM > ERRM-FS > NC 5. ARS: BD > ERRM > PRMTA > NC >> ERRM
Natu et al. [79]/2015	1. PRMTA (with additive water/propylene glycol (PG) (100/0, 80/20 and 50/50) (24 h set II) 2. NC	hDPSCs/ Indirect1	1. Attachment, viability and proliferation: MTS (D 1, 3 and 5) 2. Odonto-/Osteogenesis: ARS (D 7 and 14) and RT-PCR (D 7 and 14) 3. Migration: NM	- A: NM - M: NM	1. D1: NSD 2. D3: NC > 80/20 PRMTA > 50/50 PRMTA > 100/0 PRMTA 3. D5: NC >> 80/20 PRMTA > 100/0 PRMTA > 50/50 PRMTA	1. ALP: 1.1. D7: NC >> 80/20 PRMTA > 100/0 PRMTA > 50/50 PRMTA 1.2. D14: 80/20 PRMTA > 100/0 PRMTA > 50/50 PRMTA >> NC 2. OCN: 2.1. D7: 100/0 PRMTA > NC > 50/50 PRMTA > 80/20 PRMTA 2.2. D14: 80/20 PRMTA > 100/0 PRMTA >> 50/50 PRMTA > NC 3. Runx2: 3.1. D7: 80/20 PRMTA > 100/0 PRMTA > 50/50 PRMTA > NC 3.2. D14: 100/0 PRMTA > 50/50 PRMTA >> 80/20 PRMTA > NC 4. DSPP: 4.1. D7: NC >> 50/50 PRMTA > 100/0 PRMTA > 80/20 PRMTA 4.2. D14: 100/0 PRMTA > 80/20 PRMTA > 50/50 PRMTA >> NC
Margunato et al. [80]/2015	1. PRMTA (48 h set II) 2. BD (48 h set II) 3. MM-MTA (48 h set II) 4. Dimethyl sulfoxide (DMSO) (CP) 5. NC	hBMSCs/ Indirect2	1. Attachment, viability and proliferation: MTS (D 1, 3, 7 and 14) 2. Odonto-/Osteogenesis: RT-PCR (D14) and ALP (D14) 3. Migration: NM	- A: NM - M: NM	1. D1 and D3: NC > PRMTA >> MM-MTA > BD > CP 2. D7: MM-MTA >> PRMTA > BD = NC > CP 3. D14: BD >> MM-MTA > PRMTA > NC > CP	1. ALP activity: PRMTA >> MM-MTA > CP > BD > NC 2. COL1A: PRMTA > BD > CP > MM-MTA >> NC 3. ON: CP >> PRMTA > BD > MM-MTA >> NC 4. Runx2: PRMTA > MM-MTA > BD > CP >> NC
Shi et al. [111]/2012	1. Polymeric powder coatings (PPC) 2. White PRMTA-enriched PPC (WMPPC) 3. Gray PRMTA-enriched PPC (GMPPC) 4.NC	hBMSCs/ Direct1	1. Attachment, viability and proliferation: MTT (D 1 and 3) and CAA (D 1 and 3) 2. Odonto-/Osteogenesis: NM 3. Migration: NM	- A: 1.D1: WMPPC > GMPPC > NC > PPC 2.D3: significantly higher in GMPPC; GMPPC > WMPPC > PPC > NC - M: NM	1. D1: GMPPC = WMPPC > PPC > NC 2. D3: GMPPC > WMPPC >> PPC > NC	NM
Ong et al. [102]/2012	1. Accelerated-set white PRMTA (AWMTA) (24 h set RT) (1.5625, 3.125, 6.25, 12.5 and 25 mg/mL dilutions) 2. Accelerated-set Malaysian white PC (AMWPC) (24 h set RT) (1.5625, 3.125, 6.25, 12.5 and 25 mg/mL dilutions) 3. NC	SHED/ Indirect1	1. Attachment, viability and proliferation: MTT (D3) 2. Odonto-/Osteogenesis: NM 3. Migration: NM	- A: NM - M: NM	1. 1.5625, 3.125 and 6.25 mg/mL: NC > AMWPC > AWMTA 2. 12.5 and 25 mg/mL: NC >> AWMTA > AMWPC	NM
Liu et al. [81]/2020	1. iRFS (2 mg/mL concentration) 2. PRMTA (CP) (2 mg/mL concentration) 2.NC	SCAP/ Indirect3	1. Attachment, viability and proliferation: BrdU (20 h) and MTT (D 1, 2, 3 and 4) 2. Odonto-/Osteogenesis: qRT-PCR (D6) and ARS (D28) 3. Migration: WHA (12 and 24 h) and TMA (24 h)	- A: NM - M: WHA and TMA: iRFS > PRMTA >> NC	20 h, D1, D2, D3 and D4: NSD	1. ARS: iRFS > PRMTA >> NC 2. ALP and DSPP: iRFS > PRMTA >> NC
López-García et al. [106]/2019	1. ERRM (48 h set II) (1:1, 1:2 and 1:4 dilutions) 2. Ceraseal (48 h set II) (1:1, 1:2 and 1:4 dilutions) 3. Endoseal MTA (48 h set II) (1:1, 1:2 and 1:4 dilutions) 4.NC	hPDLSCs/ Indirect1	1. Attachment, viability and proliferation: MTT (D 1, 2 and 3) and SEM (D3) 2. Odonto-/Osteogenesis: qRT-PCR (D 3, 7, 14 and 21) and ARS (D21) 3. Migration: WHA (D 1, 2 and 3)	- A: D3: ERRM ≈ Ceraseal >> NC >> Endoseal - M: 1.D1: 1.1. 1:1 dilution: Ceraseal > ERRM > NC >> Endoseal 1.2. 1:2 dilution: ERRM > Ceraseal > NC >> Endoseal 1.3. 1:4 dilution: ERRM > NC > Endoseal = Ceraseal 2. D2: 2.1. 1:1 dilution: Ceraseal >> ERRM > NC > Endoseal 2.2. 1:2 dilution: ERRM >> Ceraseal > NC > Endoseal 2.3. 1:4 dilution: ERRM >> NC > Ceraseal > Endoseal 3. D3: 3.1. 1:1 and 1:2 dilutions: ERRM >> Ceraseal > NC > Endoseal 3.2. 1:4 dilution: ERRM > NC > Ceraseal > Endoseal	1. 1:1 dilution: 1.1. D1: ERRM > Ceraseal > NC >> Endoseal 1.2. D2: NC = Ceraseal > ERRM >> Endoseal 1.3. D3: Ceraseal > NC > ERRM >> Endoseal 2. 1:2 dilution: 2.1. D1: Ceraseal > ERRM > NC > Endoseal 2.2. D2: Ceraseal > NC > ERRM >> Endoseal 2.3. D3: ERRM > Ceraseal > NC >> Endoseal 3. 1:4 dilution: 3.1. D1: Ceraseal > ERRM > NC > Endoseal 3.2. D2: NC = Ceraseal > ERRM >> Endoseal 3.3. D3: Ceraseal > ERRM > NC >> Endoseal	1. ARS: ERRM > Ceraseal >> NC > Endoseal 2. ALP: 1.1. D3 and D7: Ceraseal >> ERRM > NC 1.2. D14 and D21: Ceraseal >> NC = ERRM
Kim et al. [82]/2020	1. PRMTA (24 h set II) 2. BD (24 h set II) 3. TCLC (24 h set II) 4. Dycal (24 h set II) 5. NC	hDPSCs/ Indirect2	1. Attachment, viability and proliferation: MTT (D 1, 2, 3 and 5) 2. Odonto-/Osteogenesis: ALP (D14) and ARS (D 7, 14 and 21) 3. Migration: WHA (D 1, 2, 3 and 4)	- A: NM - M: 1. D1, D2 and D3: NC = BD > PRMTA >> TCLC > Dycal 2. D4: NC = BD = PRMTA >> TCLC > Dycal	1. D1: NC >> PRMTA = BD > TCLC >> Dycal 2. D2, D3 and D5: NC >> BD > PRMTA > TCLC >> Dycal	1. ALP activity: 1.1. D3 and D5: Dycal > TCLC >> BD > PRMTA > NC 1.2. D7: TCLC > Dycal >> BD > PRMTA > NC 1.3. D10: BD > Dycal > TCLC > NC > PRMTA 1.4. D14: BD > NC > Dycal > TCLC > PRMTA 2. ARS: 2.1. D7: Dycal > TCLC >> PRMTA > NC > BD 2.2. D14: Dycal > TCLC > PRMTA > BD >> NC 2.3. D21: TCLC > PRMTA > Dycal > BD >> NC
Petta et al. [83]/2020	1. MTA Angelus (24 h set II) (10% concentration) 2. BD (24 h set II) (10% concentration 3. Two paste calcium hydroxide cement (CHC) (24 h set II) (10% concentration 4. Mineralization medium (CP) 5. NC	hDPSCs/ Indirect1	1. Attachment, viability and proliferation: NM 2. Odonto-/Osteogenesis: ARS (D14) 3. Migration: NM	- A: NM - M: NM	NM	ARS: BD > MTA > CHC = CP >> NC
Omidi et al. [84]/2019	1. MTA Angelus (48 h set II) (1:1 dilution) 2. BD (48 h set II) (1:1 dilution) 3. CEM (48 h set II) (1:1 dilution) 4. TCLC (48 h set II) (1:2 dilution) 5. NC	hDPSCs/ Indirect1	1. Attachment, viability and proliferation: MTT (D 1, 2 and 3) 2. Odonto-/Osteogenesis: NM 3. Migration: TMA (D1)	- A: NM - M: D1: CEM > BD >> NC > TCLC > MTA	1. D1: BD > CEM > TCLC > NC > MTA 2. D2: TCLC > CEM >> MTA > NC > BD 3. D3: TCLC > BD = CEM > MTA >> NC	NM
Collado-González et al. [107]/2017	1. GuttaFlow Bioseal (48 h set II) (1:1, 1:2 and 1:4 dilutions) 2. GuttaFlow2 (48 h set II) (1:1, 1:2 and 1:4 dilutions) 3. MTA Fillapex (48 h set II) (1:1, 1:2 and 1:4 dilutions) 4. AH Plus (48 h set II) (1:1, 1:2 and 1:4 dilutions) 5. NC	hPDLSCs/ Indirect1	1. Attachment, viability and proliferation: MTT (D 1, 2, 3 and 7) 2. Odonto-/Osteogenesis: NM 3. Migration: NM	- A: NM - M: NM	1. 1:1 dilution: 1.1. D1 and D2: NC = Bioseal = GuttaFlow2 >> Fillapex = AH 1.2. D3: NC > Bioseal = GuttaFlow2 > Fillapex = AH 1.3. D7: Bioseal >> NC = GuttaFlow2 > Fillapex = AH 2. 1:2 dilution: 2.1. D1 and D2: NC = Bioseal = GuttaFlow2 = AH >> Fillapex 2.2. D3: NC > Bioseal > GuttaFlow2 > AH > Fillapex 2.3. D7: Bioseal >> NC > GuttaFlow2 >> Fillapex > AH 3. 1:4 dilution: 3.1. D1 and D2: NC = Bioseal = GuttaFlow2 = AH >> Fillapex 3.2. D3: NC > AH > Bioseal > GuttaFlow2 >> Fillapex 3.3. D7: Bioseal >> NC = GuttaFlow2 >> AH = Fillapex	NM
Çelik et al. [86]/2020	1. PRMTA (48 h set II) (with and without RSV) 2. BD (48 h set II) (with and without RSV) 3. TCLC (48 h set II) (with and without RSV) 4. CH (48 h set II) (with and without RSV) 5. Calcimol LC (resin modified calcium hydroxide) (48 h set II) (with and without RSV) 6. NC (with and without RSV)	hBMSCs/ Indirect2	1. Attachment, viability and proliferation: MTT (4 h) 2. Odonto-/Osteogenesis: NM 3. Migration: NM	- A: 4 h: NC > CP RSV > BD + RSV > PRMTA + RSV > TCLC + RSV > BD > TCLC > PRMTA > Calcimol + RSV >> Calcimol > CH + RSV > CH - M: NM	NM	NM
Sun et al. [87]/2017	1. iRFS (24 h set II) (0.2 and 2 mg/mL concentrations) 2. BD (24 h set II) (CP) (0.2 and 2 mg/mL concentrations) 3. NC	hDPSCs/ Indirect3	1. Attachment, viability and proliferation: CCK-8 (D 1, 3 and 7) 2. Odonto-/Osteogenesis: ALP (D 7 and 14), ARS (D21) and qRT-PCR (D 1, 7 and 14) 3. Migration: WHA (D1) and TMA (D1)	- A: NM - M: WHA and TMA: 0.2 mg/mL iRFS > 2 mg/mL iRFS >> NC > 0.2 mg/mL BD > 2 mg/mL BD	1. D1 and D3: NSD 2. D7: 0.2 mg/mL BD > 0.2 mg/mL iRFS > 2 mg/mL BD = 2 mg/mL iRFS > NC	1.ALP activity: D7 and D14: 0.2 mg/mL iRFS > 2 mg/mL iRFS = 0.2 mg/mL BD > 2 mg/mL BD >> NC 2. ARS: 0.2 mg/mL iRFS >> 0.2 mg/mL BD > 2 mg/mL iRFS > NC > 2 mg/mL BD 3. COL1: 3.1. D1: NC > 2 mgiRFS > 0.2 mg/mL BD > 0.2 mg/mL iRFS > 2 mg/mL BD 3.2. D7: NC >> 0.2 mg/mL iRFS > 0.2 mg/mL BD > 2 mg/mL BD > 2 mg/mL iRFS 3.3. D14: 0.2 mg/mL iRFS >> 0.2 mg/mL BD > 2 mg/mL BD > NC > 2 mg/mL iRFS 4. OCN: 4.1. D1: 2 mg/mL iRFS > 2 mg/mL BD > NC = 0.2 mg/mL iRFS = 0.2 mg/mL BD 4.2. D7: 0.2 mg/mL iRFS >> 2 mg/mL iRFS = NC > 2 mg/mL BD > 0.2 mg/mL BD 4.3. D14: 0.2 mg/mL iRFS > 2 mg/mL iRFS >> NC > 0.2 mg/mL BD > 2 mg/mL BD
Victoria-Escandell et al. [96]/2017	1. MTA Angelus (24 h or 48 h or 1 week or 15D or 30D set II) (1:2 dilution) 2. MTA Fillapex (24 h or 48 h or 1 week or 15D or 30D set II) (1:2 dilution) 3. AH Plus (24 h or 48 h or 1 week or 15D or 30D set II) (1:2 dilution) 4. NC	hDPSCs/ Indirect1	1. Attachment, viability and proliferation: SRB (D1) 2. Odonto-/Osteogenesis: NM 3. Migration: NM	- A: NM - M: NM	1. D1: NC > Fillapex > Angelus > AH 2. D2, D7, D15 and D30: NC > Angelus > AH > Fillapex	NM
Collado-González et al. [88]/2019	1. PRMTA (1 week set II) 2. MTA Repair HP (1 week set II) 3. NC	hPDLSCs/ Indirect1	1. Attachment, viability and proliferation: MTT (D 1, 2 and 3) and SEM (D3) 2. Odonto-/Osteogenesis: NM 3.Migration: NM	- A: D3: NSD - M: NM	1. 1:1 dilution: 1.1. D1: HP > PRMTA = NC 1.2. D2 and D3: NC = HP > PRMTA 2. 1:2 dilution: 2.1. D1 and D3: HP > PRMTA > NC 2.2. D2: HP > NC > PRMTA 3. 1:4 dilution: D1, D2 and D3: HP > PRMTA >> NC	NM
Wu et al. [116]/2021	1. iRSP (72 h set II) (0.02, 0.2, 2, 5 and 10 mg/mL concentrations) 2. NC	SCAP/ Indirect3	1. Attachment, viability and proliferation: CCK-8 (D 1, 3 and 5) 2. Odonto-/Osteogenesis: ALP (D 3, 7 and 14), ARS (3, 7, 14 and 21) and qRT-PCR (D 3 and 7) 3. Migration: WHA (12 h)	- A: NM - M: 12 h: 0.2 mg/mL iRSP > 0.02 mg/mL iRSP > 2 mg/mL iRSP >> NC	D1, D3 and D5: 0.2 mg/mL iRSP >> 2 mg/mL iRSP > 0.02 mg/mL iRSP > NC >> 5 mg/mL iRSP > 10 mg/mL iRSP	1. ALP activity: 0.2 mg/mL iRSP > 2 mg/mL iRSP > 0.02 mg/mL iRSP >> NC 2. ARS: 0.2 mg/mL iRSP >> NC 3.OCN, OSX, Runx2 and DSPP: 0.2 mg/mL iRSP >> NC
Manaspon et al. [41]/2021	1. PRMTA (24 h set RT) (10%, 25%, 50% and 100% concentrations) 2. BD (24 h set RT) (10%, 25%, 50% and 100% concentrations) 3.TCLC (24 h set RT) (10%, 25%, 50% and 100% concentrations) 4. Dycal (24 h set RT) (10%, 25%, 50% and 100% concentrations) 5. NC	hDPSCs/ Direct1 (SEM) and Indirect1 (MTT)	1. Attachment, viability and proliferation: MTT (D 1, 4 and 7) and SEM (3 h, 6 h, 24 h and 48 h) 2. Odonto-/Osteogenesis: ALP (D14), ARS (D14) and RT-PCR (D 4 and 10) 3. Migration: WHA (D1)	- A: PRMTA ≈ BD ≈ NC >> Dycal ≈ TCLC - M: PRMTA ≈ BD >> NC >> Dycal ≈ TCLC	10%, 25%, 50% and 100% concentrations: PRMTA ≈ BD >> NC >> Dycal ≈ TCLC	1. ALP activity and ARS: BD > PRMTA >> NC 2. Runx2: 2.1. D4: BD >> NC > PRMTA 2.2. D10: PRMTA > BD >> NC 3. DMP1: D4 and D10: BD > PRMTA >> NC 4. DSPP: 4.1. D4: PRMTA > BD >> NC 4.2. D10: BD > PRMTA >> NC 5. OCN: 5.1. D4: BD >> NC > PRMTA 5.2. D10: PRMTA > BD >> NC
Chung et al. [97]/2019	1. PRMTA + LPS (24 h set II) 2. Retro MTA + LPS (24 h set II) 3. BD + LPS (24 h set II) 4. Dycal + LPS (24 h set II) 5. NC 6. LPS	hDPSCs/ Indirect3	1. Attachment, viability and proliferation: CCK-8 (D 1 and 2) 2. Odonto-/Osteogenesis: qRT-PCR (12 h, D1 and D2) 3. Migration: NM	- A: NM - M: NM	1.D1: NC >> Retro + LPS > BD + LPS > PRMTA + LPS > LPS = Dycal + LPS 2. D2: NC >> BD + LPS > Retro + LPS > LPS > PRMTA + LPS > Dycal + LPS	1. ALP: 1.1. 12 h and D1: Dycal + LPS >> Retro + LPS > BD + LPS > PRMTA + LPS > LPS > NC 1.2. D2: Dycal + LPS >> NC > Retro + LPS > LPS > BD + LPS > PRMTA + LPS 2. OCN: 2.1. 12 h: BD + LPS > Retro + LPS > Dycal + LPS > PRMTA+ LPS > LPS > NC 2.2. D1: Dycal + LPS >> Retro + LPS > PRMTA + LPS > BD + LPS > NC 2.3. D2: NC >> Dycal + LPS > BD + LPS > Retro + LPS > LPS > PRMTA + LPS 3. Runx2: 3.1. 12 h: NC > Dycal + LPS >> BD + LPS > Retro + LPS > PRMTA + LPS > LPS 3.2. D1: Dycal + LPS >> NC > Retro + LPS > BD + LPS > PRMTA + LPS > LPS 3.3. D2: Dycal + LPS > NC >> Retro + LPS > LPS > BD + LPS > PRMTA + LPS
Birant et al. [89]/2020	1. PRMTA (24 h set II) 2. NeoMTA Plus (24 h set II) 3. BD (24 h set II) 4. NC	hDPSCs/ Indirect1	1. Attachment, viability and proliferation: FCM (D 1, 3 and 7) 2. Odonto-/Osteogenesis: NM 3. Migration: NM	- A: NM - M: NM	1. D1: NC >> BD > NeoMTA > PRMTA 2. D3: NC >> BD > PRMTA > NeoMTA 3. D7: BD >> PRMTA > NeoMTA > NC	NM
Sanz et al. [90]/2021	1. BD (48 h set II) (1:1, 1:2 and 1:4 dilutions) 2. TCPT (48 h set II) (1:1, 1:2 and 1:4 dilutions) 3. TCLC (48 h set II) (1:1, 1:2 and 1:4 dilutions) 4. NC	hDPSCs/ Indirect1	1. Attachment, viability and proliferation: MTT (D 1, 2 and 3) and SEM (D3) 2. Odonto-/Osteogenesis: RT-PCR (D14) and ARS (D21) 3. Migration: WHA (D 1, 2 and 3)	- A: D3: BD >> NC = TCPT > TCLC - M: 1. 1:1 dilution: 1.1. D1: NC >> BD > TCPT > TCLC 1.2. D2 and D3: NC >> TCPT > BD > TCLC 2. 1:2 dilution: 2.1. D1 and D2: NC > BD > TCPT > TCLC 2.2. D3: NC = BD > TCPT > TCLC 3. 1:4 dilution: 3.1. D1: BD >> NC > TCPT > TCLC 3.2. D2: TCPT >> NC = BD > TCLC 3.3. D3: NC = BD = TCPT > TCLC	1. 1:1 and 1:2 dilutions: NC >> BD > PT > LC 3. 1:4 dilution: 3.1. D1: TCPT > NC > BD > TCLC 3.2. D2: BD > NC > TCPT > TCLC 3.3. D3: NC > BD > TCPT > TCLC	1. ARS: BD > TCPT >> TCLC = NC 2. DSPP: BD > TCPT >> NC 3. Runx2: TCPT > BD >> NC 4. ALP: NC >> TCPT > BD 5. COL1A1: NC > TCPT > BD 6. ON: TCPT >> NC > BD
Rahimi et al. [98]/2019	1. PC (24 h set II) 2. PC + ZnO (24 h set II) 3. PC + ZrO_2_ (24 h set II) 4. NC	hDPSCs/ Direct2	1. Attachment, viability and proliferation: MTT (D 7, 14 and 21) 2. Odonto-/Osteogenesis: ALP (D 7, 14 and 21) 3. Migration: NM	- A: NM - M: NM	1. D7: PC + ZrO_2_ >> PC > NC = PC + ZnO 2. D14: PC >> PC + ZnO > PC + ZrO_2_ > NC 3. D21: PC + ZrO_2_ = PC + ZnO > PC > NC	ALP activity: 1. D7: PC + ZnO >> PC + ZrO_2_ = PC > NC 2. D14: PC = PC + ZnO >> PC + ZrO_2_ > NC 3.D21: NSD
Rodríguez-Lozano et al. [100]/2015	1. MTA Fillapex (48 h set II) (1:1, 1:2 and 1:4 dilutions) 2. AH Plus (48 h set II) (1:1, 1:2 and 1:4 dilutions) 3. TF BC (48 h set II) (1:1, 1:2 and 1:4 dilutions) 4. NC	hDPSCs/ Indirect1	1. Attachment, viability and proliferation: MTT (D 1, 2 and 3) and SEM (D4) 2. Odonto-/Osteogenesis: NM 3. Migration: WHA (D 1 and 2)	- A: 1:1, 1:2 and 1:4 dilutions: TF BC >> NC > AH > Fillapex - M: 1.D1: BC > AH >> NC >> Fillapex 2.D2: NC = AH = TF BC >> Fillapex	1:1, 1:2 and 1:4 dilutions: 1. D1: TF BC = AH = NC >> Fillapex 2. D2 and D3: TF BC = NC >> AH > Fillapex	NM
Jaberiansari et al. [91]/2014	1. PRMTA (48 h set II) (1:2 dilution) 2. MTA Angelus (48 h set II) (1:2 dilution) 3. CEM (48 h set II) (1:2 dilution) 4. NC	hDPSCs/ Indirect1	1. Attachment, viability and proliferation: MTT (D 1, 2 and 3) 2. Odonto-/Osteogenesis: NM 3. Migration: WHA (D 1 and 2)	- A: NM - M: NM	1. D1 and D2: CEM > Angelus > PRMTA >> NC 2. D3: PRMTA > CEM > Angelus >> NC	NM
Loison-Robert et al. [92]/2018	1. BD (24 h set II) 2. BioRoot RCS (24 h set II) 3. NC	hDPSCs/ Direct2	1. Attachment, viability and proliferation: MTT (D 1, 5 and 8) 2. Odonto-/Osteogenesis: ARS (D10) and qRT-PCR (D7) 3. Migration: WHA (D 1, 2 and 7)	- A: NM - M: D1 and D2: NC >> BD > RCS	D1, D5 and D8: NC >> BD = RCS	1. ARS: BD ≈ RCS >> NC 2. ALP and OPN: NC >> BD ≈ RCS 3. Runx2: RCS > BD >> NC
Sun et al. [101]/2021	1. ERRM (48 h set II) 2. NeoPutty 3. NC	hDPSCs/ Indirect2	1. Attachment, viability and proliferation: NM 2. Odonto-/Osteogenesis: ALP (week 1, 2 and 3), ARS (weeks 1, 2 and 3) and qRT-PCR (week 1, 2 and 3) 3. Migration: NM	- A: NM - M: NM	NM	1. ALP activity: weeks 1, 2 and 3: ERRM > NeoPutty >> NC 2. ARS: 2.1. week 1: NSD 2.2. weeks 2 and 3: NeoPutty > ERRM >> NC 3. Runx2: 3.1. weeks 1, 2: ERRM > NeoPutty >> NC 3.2. week 3: NeoPutty > ERRM >> NC 4. OSX: 4.1. weeks 1, 2: ERRM > NeoPutty >> NC 4.2. week 3: NC > NeoPutty > ERRM 5. DSPP: weeks 1, 2 and 3: ERRM > NeoPutty >> NC 6. OCN: 6.1. week 1: ERRM > NeoPutty >> NC 6.2. week 2: ERRM = NeoPutty >> NC 6.3. week 3: NeoPutty >> NC > ERRM 7. DMP1: 7.1. week 1: NeoPutty > NC > ERRM 7.2. week 2: NeoPutty > ERRM > NC 7.3. week 3: NeoPutty >> ERRM = NC 8. BSP: 8.1. week 1: NSD 8.2. week 2: ERRM >> NC > NeoPutty 8.3. week 3: ERRM > NeoPutty >> NC 9. ALP: 9.1. weeks 1 and 2: NeoPutty > ERRM >> NC 9.2. week 3: NeoPutty > NC > ERRM
Kim et al. [93]/2021	1. PRMTA (48 h set II) 2. BD (48 h set II) 3. TCLC (48 h set II) 4. Dycal (48 h set II) 5. NC	hDPSCs/ Indirect1	1. Attachment, viability and proliferation: CCK-8 (D 1, 2, 4 and 6) 2. Odonto-/Osteogenesis: ALP (D 3 and 6) and qRT-PCR (D 9 and 14) 3. Migration: NM	- A: NM - M: NM	1. D1 and D2: NSD 2. D4 and D6: BD > PRMTA >> NC > TCLC >> Dycal	1. ALP activity: TCLC = Dycal >> BD = PRMTA > NC 2.R unx2: TCLC > PRMTA > Dycal > BD >> NC 3. OCN: TCLC = PRMTA >> NC > Dycal > BD 4. OPN:NC >> Dycal > PRMTA > TCLC = BD 5. DMP1: 5.1. D9: PRMTA > Dycal > TCLC > BD >> NC 5.2. D14: NC > Dycal > PRMTA > TCLC = BD 6. DSPP: 6.1. D9: NC >> PRMTA = Dycal > TCLC = BD 6.2. D14: PRMTA > BD >> NC = Dycal = TCLC
Assadian et al. [92]/2022	1. Ortho MTA (OMTA) (24 h set II) (10%, 25%, 50% and 100% concentrations) 2. BD (24 h set II) (10%, 25%, 50% and 100% concentrations) 3. CEM (24 h set II) (10%, 25%, 50% and 100% concentrations) 4. NC	hDPSCs/ Indirect1	1. Attachment, viability and proliferation: MTT (D 1, 3 and 5) 2. Odonto-/Osteogenesis: RT-PCR (D 7 and 14) 3. Migration: NM	- A: NM - M: NM	D1, D3 and D5: NSD	1. DSPP: OMTA >> BD > NC > CEM 2. DMP1: BD >> OMTA > CEM > NC

**Abbreviations: BA:** Bioaggregate, **BD:** Biodentine, **CEM:** calcium-enriched matrix, **CH:** calcium hydroxide, **DH:** dried heat, **FM:** freshly mixed, **hBMSCs:** human bone marrow stem cells, **hDPSCs:** human dental pulp stem cells, **hPDLSCs:** human periodontal ligament stem cells, **hTGSCs:** human tooth germ stem cells, **hUVESCs:** human umbilical vein endothelial cells, **II:** in incubation, **iRBP:** iRoot BP, **iRFS:** iRoot fast set, **IRM:** intermediate restorative material, **iRSP:** iRoot SP, **NC:** negative control group (untreated cells), **OMTA:** OrthoMTA, **PC:** Portland cement, **PRMTA:** ProRoot MTA, **rBMSCs:** rat bone marrow stem cells, **rDPSCs:** rat dental pulp stem cells, **RT:** room temperature, **SCAP:** stem cells from apical papilla, **SHED:** stem cells from human exfoliated deciduous teeth, **TCLC:** TheraCal LC, **TCPT:** TheraCal PT, **TF:** TotalFill, and **WRST:** well root ST.

**Table 3:** All 3 of the included in vivo studies, their tested and control groups, interventions, methods of assessment, evaluation periods and results.

Author/Year	Cements and Materials (Setting Times and Conditions) (Dilutions/Concentrations)	Cells/ Interventions	Methods of Assessment	Results Attachment (A)/Viability/Odonto-/ Migration (M)Proliferation Osteogenesis		
Jeanneau et al. [45]/2017	1. BD (FM) 2. TCLC (FM) 3. NC	hDPSCs/ Direct3	1. Attachment, viability and proliferation: MTT (D 1, 3 and 5) 2. Odonto-/Osteogenesis: NM 3. Migration: NM	- A: NM - M: NM	D1, D3 and D5: NC >> BD >> TCLC	NM
Abedi-Amin et al. [95]/2017	1. Experimental PC (Exp. PC) (24 h set II) 2. PC (24 h set II) (CP) 3. Two light curing cements: LC-CaP (24 h set II) and LC-Si/CaP (24 h set II) 4. NC	hDPSCs/ Direct4	1. Attachment, viability and proliferation: MTS (D 1, 2, 4 and 7) 2. Odonto-/Osteogenesis: NM 3. Migration: NM	- A: NM - M: NM	D1, D4 and D7: NC >> LC-CaP > LC-Si/CaP >> Exp. PC >> PC	ALP activity: Exp PC > PC >> LC-CaP > LC-Si/CaP
Birant et al. [85]/2021	1. PRMTA (FM or 24 h set RT) 2. MTA Fillapex (FM or 24 h set RT) 3. MTA Angelus (FM or 24 h set RT) 4. CEM (FM or 24 h set RT) 5. NC	hDPSCs/ Direct4	1. Attachment, viability and proliferation: SEM (D7) 2. Odonto-/Osteogenesis: NM 3. Migration: NM	- A: 1. CEM: Adhesion was seen in both 24 h set and FM groups 2. MTA Fillapex: Adhesion was seen only in the 24 h set group 3. MTA Angelus and PRMTA: Adhesion was seen only in the FM group - M: NM	NM	NM

**Abbreviations: BD:** Biodentine, **CEM:** calcium-enriched matrix, **FM:** freshly mixed, **hDPSCs:** human dental pulp stem cells, **II:** in incubation, **NC:** negative control group (untreated cells), **PC:** Portland cement, **PRMTA:** ProRoot MTA, **RT:** room temperature, and **TCLC:** TheraCal LC.

#### 2.2.3. Types of Interventions

**Figure 4:**
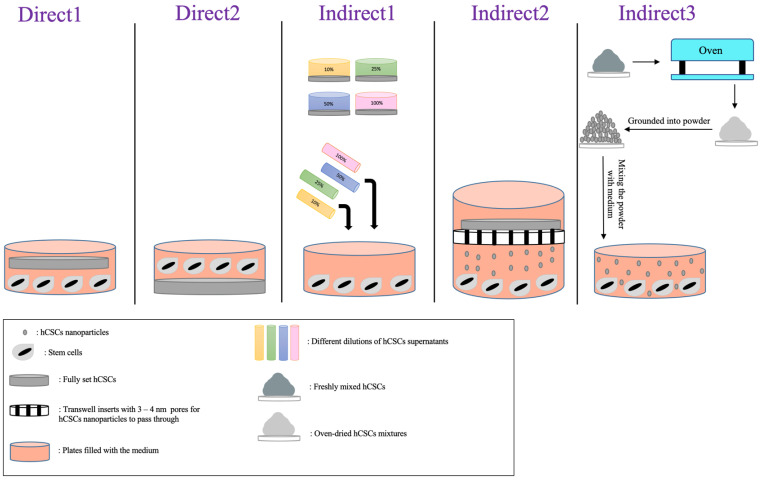
Visual description of direct and indirect exposure methods assessed in in vitro studies.

**Figure 5:**
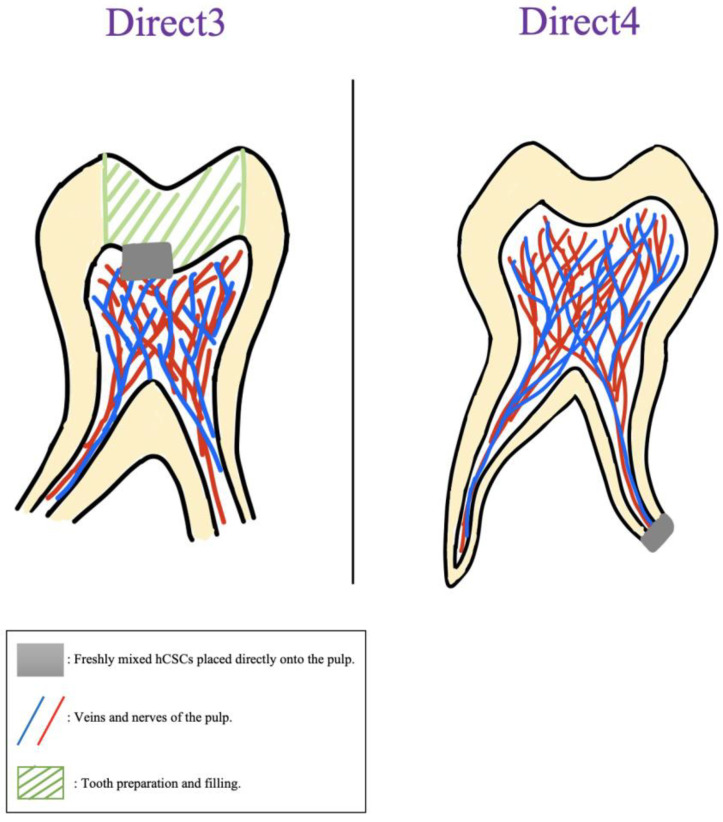
Visual description of direct exposure methods assessed in in vivo studies.

The different approaches that the authors used to place cells in contact with the materials were categorized into two major groups: direct contact and indirect contact. Furthermore, each group had different approaches, which are all displayed in Table 4 with their descriptions. Figure 4 and Figure 5 showcase a visual description of all of the exposure methods in in vitro and in vivo studies, respectively.

Untreated stem cells were considered as a negative control group in all of the studies and all of the variables of the cements were analyzed in comparison to them. For an easier and more convenient way of comparing different outcomes, the following abbreviations were constructed:

Outcomes that were significantly better and/or statistically higher than NC: significantly higher (SH).

Outcomes that showed no significant difference with NC: no significant difference (NSD).

Outcomes that were significantly worse and/or statistically lower than NC: significantly lower (SL).

**Figure 6:**
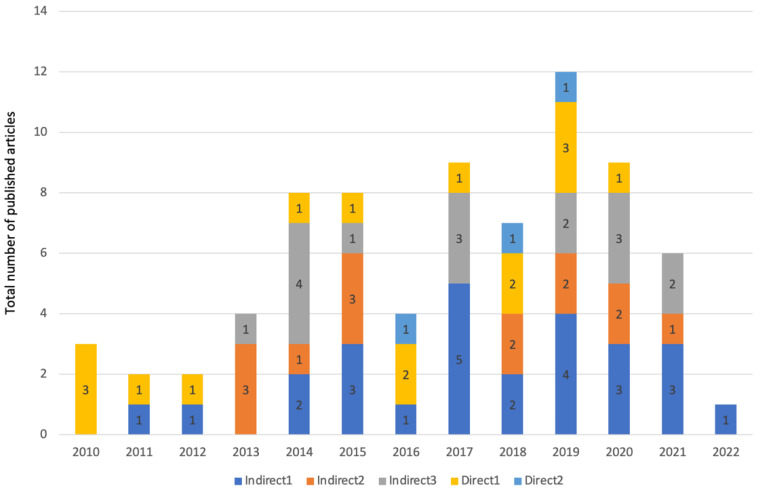
Total number of included in vitro studies in this review from each year, along with the number of articles that have assessed each of the five direct and indirect exposure methods in vitro.

In addition, Figure 6 showcases a visual description of the assessment frequency of different direct and indirect exposure methods in the included studies from 2010 to 2022.

#### 2.2.4. Methods of Assessment

##### Viability and Proliferation

Cellular viability and proliferation were examined in a total of 69 articles, using the following assays and methods (Supplementary Table S1): MTT, LDA, SEM, MTS, CCK-8, XTT, ELISA, Prostoblue, BrdU, WST-1, FCM, cell lysates, Alamar Blue, CLSM, SRB, CyQuant^TM^ assay, IF, WB and OZBlue assay.

##### Attachment

Cellular attachment was examined in a total of 27 studies. Attachment was tested using the following assays and methods: SEM, MTT, LDA, Prostoblue, ELISA, CLSM, CAA, WST-1, PCM and BrdU.

##### Migration

Cellular migration was examined in a total of 23 studies. Migration was tested using the following assays and methods: WHA, TMA, RT-PCR and Cell Tracker^TM^ Green CMFDA.

##### Odonto-/Osteogenesis

Alkaline phosphatase (ALP) activity was examined in a total of 25 studies using the ALP activity assay kit (colorimetric). Alizarin red staining (ARS) was assessed in a total of 22 studies. Gene expression was examined in a total of 39 studies using RT-PCR or qRT-PCR.

### 2.3. Results of Individual Studies

#### 2.3.1. In Vivo Studies

Out of the three included vivo studies, none of them examined cellular migration, mineralization (ARS), or gene expressions. Only one study examined ALP activity, however, it did not compare the results of the cements with the NC group [95]. Two of the in vivo studies investigated the viability/proliferation abilities of their hCSCs and in both of them NC showed SH results [45,95]. Only one study examined cellular attachment and reported that CEM showed cellular adhesion in both FM and 24 h set RT conditions (Table 3).

#### 2.3.2. Setting Times and Conditions In Vitro

The 24 h setting in incubation (II) technique was the most used approach and had remarkable rates of SH results, while most of the cases of FM cements led to SL results compared to NC (Table 2 and Figure 3). Compared to the 24 h II technique (n = 28), the dried heat (DH) condition was used in only four studies before the application of hCSCs. However, all of the reported biocompatibility and regenerative outcomes were similar (NSD) to the NC group or significantly better than NC (SH) [55,56,75,76]. Out of the four studies that examined the DH technique, only one of them reported their exact environment and conditions—a 50 °C oven for 15 min [53]—but the remaining three studies did not specify their environments. A total of four studies used RT as their only setting condition for hCSCs and their results were a mixture of SH, NSD, and SL outcomes compared to the NC [41,43,54,116] (Figure 3).

#### 2.3.3. Comparison of Different hCSCs In Vitro

**Figure 7:**
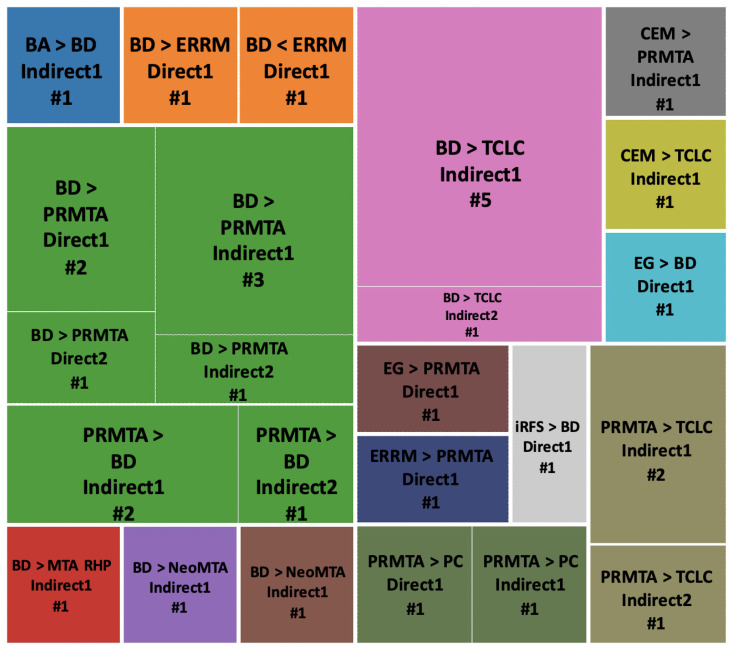
Viability/proliferation outcome comparisons of hCSCs used in different exposure methods. Abbreviations of included hCSCs: **BA:** Bioaggregate, **BD:** Biodentine, **EG:** Emdogain, **iRFS:** iRoot fast set, **PC:** Portland cement, **PRMTA:** ProRoot MTA, and **TCLC:** TheraCal LC.

**Figure 8:**
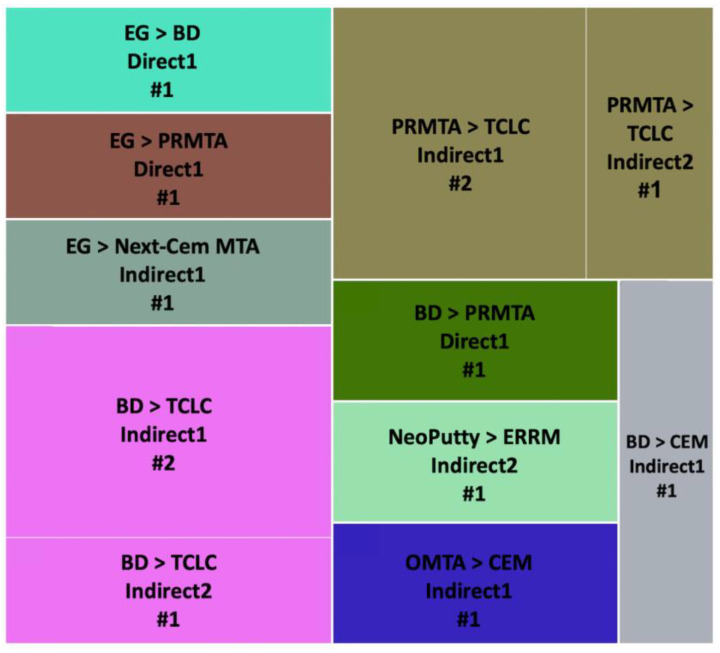
Odontogenesis outcome comparisons of hCSCs used in different exposure methods. Abbreviations of included hCSCs: **BD:** Biodentine, **EG:** Emdogain, **OMTA:** OrthoMTA, **PRMTA:** ProRoot MTA, and **TCLC:** TheraCal LC.

**Figure 9:**
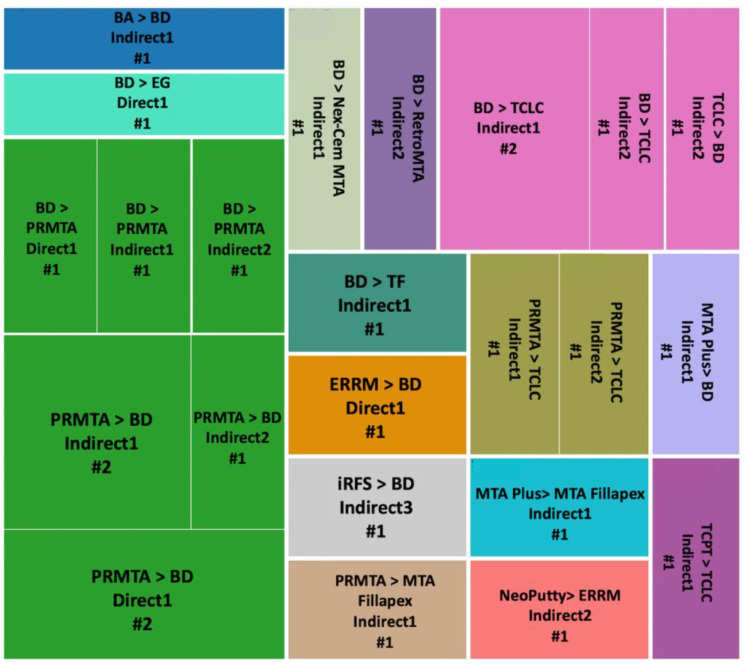
Osteogenesis outcome comparisons of hCSCs used in different exposure methods. Abbreviations of included hCSCs: **BA:** Bioaggregate, **BD:** Biodentine, **EG:** Emdogain, **iRFS:** iRoot fast set, **PRMTA:** ProRoot MTA, **TCLC:** TheraCal LC, **TCPT:** TheraCal PT, and **TF:** TotalFill.

To better comprehend the outcomes of different cements used in different approaches for each category of results (i.e., proliferation, odontogenesis, and osteogenesis), we designed three figures: Figure 7 (viability/proliferation), Figure 8 (odontogenesis), and Figure 9 (osteogenesis), to simplify the results. We only focused on the outcomes that showed significant differences between hCSCs.

##### Viability/Proliferation, Migration and Attachment

BD versus PRMTA was the most repeated comparison (n = 10), and BD showed significantly better results in five of the comparisons in Indirect1, Indirect2, Direct1 and Direct2 methods [49,51,53,72,77,78,80,84,89] (Figure 7). BD versus TCLC hCSCs were the second most compared (n = 6), and BD always showed significantly better results in both Indirect1 and Indirect2 methods [41,53,82,84,90,93]. PRMTA also showed significantly better results than TCLC (n = 3) in both Indirect1 and Indirect2 methods [41,82,93]. Additionally, PRMTA showed significantly better results than PC (n = 2) in Direct1 and Indirect1 methods [52,67] (Figure 7).

##### Odontogenesis

PRMTA versus TCLC (n = 3) and BD versus TCLC (n = 3) were the most repeated comparisons. In all of the experiments, PRMTA and BD showed significantly better results in Indirect1 and Indirect2 methods [37,93] (Figure 8).

##### Osteogenesis

BD versus PRMTA (n = 8) was the most repeated comparison, with PRMTA showing significantly better results than BD in five of the experiments in Indirect1, Indirect2 and Direct1 methods [51,57,72,77,80] (Figure 9). In BD versus TCLC (n = 4) and PRMTA versus TCLC (n = 2), TCLC always showed significantly weaker results in Indirect1 and Indirect2 methods [37,90], except for one experiment in which TCLC showed significantly better results than BD in the Indirect2 method [93] (Figure 9).

#### 2.3.4. Comparison of Different Exposure Methods In Vitro

A detailed comparison of only the SH results of all five different exposure methods is shown in Table 5. However, in terms of NSD and SL results, the outcome differences are discussed in each of the categories below.

##### Viability and Proliferation

Indirect methods performed much better, with Indirect1 having the highest rate of SH results. Direct2 had the worst performance.

##### Cellular Attachment

Direct2 was not examined in this category. Indirect3 showed SH results in all of its experiments. Indirect2 had the weakest performance with no SH outcomes.

##### Cellular Migration

Direct2 showed SL results in all of its experiments. Direct1 and Indirect3 had the highest rates of SH results.

##### ALP Activity

Indirect2 and Indirect3 both had SH results in all of their experiments. Indirect1 had higher rates of SH results compared to Direct1. Direct2 had the weakest performance, with 100% NSD results.

##### Mineralization

Direct2, Indirect2 and Indirect3 all had 100% SH results. Indirect1 had better results than Direct1.

##### ALP Expression

Indirect2 had 100% SH results, followed by Indirect1 (77%) and Indirect3 (70%). Direct methods had significantly weaker results, with Direct2 having 100% SL results and Direct1 having only 9.09% SH results.

##### Runx2 Expression

Direct2 (100%), Indirect1 (90.9%) and Indirect2 (90%) had highest rates of SH results, followed by Indirect3 (62.5%). Direct1 (33.33% SH) had the weakest performance.

##### DSPP Expression

Indirect3 (100%) and Indirect2 (83.33%) had the highest rates of SH results, followed by Indirect2 (83.33%) and Direct1 (56.25%). Direct2 was not examined in this category.

##### DMP1 Expression

Indirect1, Indirect2 and Indirect3 all had 100% SH results, while Direct1 had only 50%. Direct2 was not examined in this category.

##### OCN Expression

Indirect3 had the best performance, with 92.3% SH results, followed by Indirect1 (85.7%), Indirect2 (80%) and Direct1 (66.66%). Direct2 was not examined in this category.

##### COL1 Expression

Direct1, Indirect2 and Indirect3 all had 100% SH results, while Indirect1 only had 57.14%. Direct2 was not examined in this category.

##### BSP Expression

Direct1, Indirect1, Indirect2 and Indirect3 all had 100% SH results. Direct2 was not examined in this category.

##### OPN Expression

Indirect3 had the best performance, with 100% SH results, while Direct1 had only 25%. Both Direct2 and Indirect1 had 100% SL results. Direct2 was not examined in this category.

##### ON Expression

Indirect2 had 100% SH results, followed by Indirect1 (75%). Direct1 had 100% NSD results. Direct2 and Indirect3 were not examined in this category.

### 2.4. Summary of Outcomes of In Vitro Studies

We summarized all of the outcomes for the five different contact approaches in vitro (i.e., Direct1, Direct2, Indirect1, Indirect2, and Indirect3) into one table (Table 5). Different approaches are categorized into four groups based on their performance: (1) more than 80% of results were SH than NC; (2) 50% to 80% of results were SH than NC; (3) 33% to 50% of results were SH than NC; (4) less than 33% of results were SH than NC. Approaches that did not have even a single case of SH results were not included in Table 5.

### 2.5. Risk of Bias Assessment

The results of risk of bias assessments for in vitro studies and in vivo studies are displayed in Figures S1 and S2, respectively. The risk of bias was unclear for all three included in vivo studies. Out of the 75 in vitro studies, all of them had unclear risk of bias in the first three questions that represent the randomization of studies; however, all of them had low risk of bias in the remaining five questions of the questionnaire. Overall, all 75 in vitro studies had a low to unclear risk of bias.

### 2.6. Discussion

This systematic review was conducted to assemble all of the different direct and indirect contacts between various hCSCs and stem cells in vitro and in vivo. As mentioned in our results, there was a significant difference between the number of in vitro and in vivo studies (75 in vitro versus 3 in vivo). Amongst the five different direct and indirect approaches in vitro, indirect ones significantly outshone the direct methods in almost all different outcome categories. Indirect1 was the most used approach amongst all included studies (Table 3). Most of the studies allowed hCSCs to set for 24 h in incubators (II). PRMTA and BD were the most frequently used hCSCs and showed significantly better biological behavior (i.e., cell viability/proliferation, attachment, migration, mineralization, odonto-/osteogenesis, and variant gene expressions) compared to other utilized cements in different exposure methods (i.e., Direct1, Direct2, Indirect1, Indirect2, and Indirect3).

In our systematic electronic search, we found four systematic reviews similar to our review. Although these reviews have analyzed similar categories of outcomes to our review, they have only focused on a very small group of hCSCs, have included only a certain type of stem cell, or had chosen only direct contact [112,114,117,118]. On the contrary, these limitations were not considered in our systematic review, enabling us to compare and discuss commercially available hCSCs more comprehensively. Additionally, the categorization of all the different direct and indirect exposure methods both in vitro and in vivo has never been conducted before.

Regarding the performance modality of hCSCs, the alkaline pH of hCSCs in contact with stem cells persuades the hastiness of the carbonated apatite layer between the cement and the dentine–pulp complex [18,19]. hCSCs initiate the remineralization of tooth tissues by an epithelial–mesenchymal Bmp/Wnt-signaling complex network including mesenchymal Bmp7 [20]. hCSCs also upregulate a series of signaling transduction pathways (e.g., Wnt/β-catenin, NF-κB, MAPK family (and its subfamilies ERK, p38, and JNK), TFG-β/Smad, and P13K/AKT/mTOR) as mediators in the process of hDPSC differentiation and their odonto-/osteogenesis abilities [21,22,23,24,25,26].

#### 2.6.1. hCSCs Differences

Calcium hydroxide deposition after hCSCs hydration is pivotal to initiate the consequential biologic reactions of hCSCs in contact with stem cells [119]. Previous studies showed that both PRMTA and BD fulfill their calcium hydroxide deposition [120]. An alkaline environment is crucial for inducing proliferation and odonto-/osteogenesis by hCSCs [121]. Different studies have reported that both PRMTA and BD induce alkaline pH in contact with cells, regardless of the evaluation periods [122,123]. Furthermore, previous findings showed that BD and PRMTA have similar cytocompatibilities [124]. BD and PRMTA both have tricalcium silicate (Ca_3_SiO_5_) and dicalcium silicate (Ca_2_SiO_4_) as their major components. Additionally, BD contains calcium carbonate (CaCO_3_) (filler material) and calcium oxide (CaO) (traces), whereas PRMTA contains calcium sulfate dihydrate (CaSO_4_·2H_2_O) (filler material) and tricalcium aluminate (Ca_3_Al_2_O_6_) (traces) [125]. The reported data suggest that calcium sulfate dihydrate and calcium carbonate help PRMTA and BD, respectively, to be more cytocompatible for hDPSCs [126]. All of the mentioned reported outcomes corroborate our findings that BD and PRMTA have very similar abilities and both result in similar viability/proliferation and odonto-/osteogenesis outcomes.

#### 2.6.2. Setting Times and Conditions

Most of our included in vitro studies allowed their cements to set in incubation (II) for at least 24 h before application. The majority of these studies saw similar outcomes (NSD) with the NC group, while some of them reported even better outcomes (SH) than NC. The 37 °C, 5% CO_2_ and 95% humidity atmosphere supplied by the incubators simulates the elution of hCSC toxins in vitro and consequently prevents the damages that hCSC toxins can cause to hDPSCs, hPDLSCs and SCAP [106]. Therefore, when cements are applied immediately after mixing (freshly mixed (FM)), the biocompatibility and odonto-/osteogenesis outcomes are significantly lower (SL) and weaker than stem cells with no hCSCs (negative control (NC) group), because the hCSC toxins did not have any time to be released prior to application [48]. Additionally, some studies have reported that freshly mixed (FM) hCSCs are so toxic for stem cells that almost all of the cells were dead at the assessed evaluation periods and no cellular proliferation was observed [37]. Even when studies reported that freshly mixed hCSCs did not kill the stem cells, the odonto-/osteogenesis outcomes (e.g., DSPP gene expression, ALP gene expression, etc.) were significantly lower than NC [47]. The remarkably low number of studies using the DH or RT setting condition techniques, along with the lack of information regarding the environmental details of the DH technique, makes their current reported outcomes unreliable. Further investigations both in vitro and in vivo can examine the superiority/inferiority of the DH and RT techniques compared to II.

#### 2.6.3. Direct and Indirect Approaches In Vitro

The Indirect1 approach benefits from an adequate setting time for cements (mostly in incubation (II) for 24 h), which releases the majority of toxins before making the dilution/supernatant [101]. The medium, in direct contact with the fully set cements, spends a considerable amount of time in the incubator to make sure all of the biocompatibility and regeneration-inducing molecules are released into the medium to make an hCSC-enriched supernatant. The incubated supernatant is not only rich enough in hCSCs-inducing molecules, but it also does not have the toxicity of cements in direct contact with stem cells, and this is why Indirect1 was so successful in not only keeping cells viable, but also inducing the proliferation and regeneration in stem cells significantly better (SH) than the NC group. Indirect2 had the most versatile SH outcomes (> 80%) across all of the different examinations and was mentioned as one of the most desirable approaches in 10 of the 14 mentioned outcome measures in Table 5. The Indirect2 method required specifically designed Transwell™ permeable inserts with extremely small pores (0.3–0.4 μm) incorporated into them. In this technique, cells were on the bottom of the plates and only in indirect contact with hCSCs through the shared medium. Most of the studies that used the Indirect2 technique allowed their cements to fully set (24 h) before placing them in the Transwell inserts. The very small pores led to a very slow release of hCSC molecules into the shared medium with the stem cells. Stem cells had enough time to respond to the hCSC chemicals without being exposed to a huge amount of toxic freshly mixed cements. Hence, this approach produced a wide range of successful outcomes throughout almost all of its examinations (Table 5).

Despite the fact that Indirect1 and Indirect2 mostly resulted in significantly remarkable results, when cements are freshly mixed, the results were SL than the NC group [47,50]. These findings help us to comprehend the fact that choosing the type of contact and the setting time/condition are equally crucial for having the most remarkable outcomes. Indirect3, similar to Indirect2, appeared in 9 of the 14 examined outcomes in Table 5 as one of the approaches with the highest rates of SH results (>80%). hCSCs in this technique were ground into powder immediately after mixing (freshly mixed (FM)) and then they were dried and at the end mixed with the medium. As our collected data show, all three different indirect approaches had remarkably better outcomes than the two different direct approaches amongst all categories of outcomes. However, it is important to mention that the number of studies that each approach was used in was outstandingly different. Indirect1 had relatively lower rates of SH (>80%) results compared to the other two indirect approaches, yet Indirect1 was used in 24 studies, which is significantly more than Indirect2 and Indirect3 (12 and 16, respectively). The exceedingly high number of studies that used Indirect1 provides its outcomes with a significant level of reliability. On the other hand, the outstanding performance of Indirect2 and Indirect3 could not be ignored. Therefore, regarding the most successful indirect contact between hCSCs and cements in vitro, Indirect1 could be a solid and safe choice, with many studies reporting similarly positive outcomes, while Indirect2 and Indirect3 have shown remarkable outcomes but have been used in significantly fewer studies.

The number of experiments in each category examining each approach (direct or indirect) was significantly different. In some cases, studies with different approaches had similar results, and because they were assessed a similar number of times, their outcomes were perfectly comparable and all of them had the same level of reliability (e.g., all three different indirect approaches had high rates of SH results in ALP activity and were assessed a similar number of times). On the other hand, some of these immense differences resulted in unreliability when an approach was used in very limited studies. Some of the outcomes that were reported in a very limited number of studies were as follows: (1) Indirect3 was assessed for cellular attachment in only 2 studies, whereas, on the other hand, Indirect1 was assessed 21 times; (2) for cellular migration, Indirect3 and Direct1 both had the highest rates of SH results. However, Indirect3 was used 12 times and Direct1 only 5; (3) when assessed for mineralization, Indirect2, Indirect3 and Direct2 all showed remarkably high rates of SH results, but Direct2 was used only 2 times, and Indirect2 and Indirect3 were used 7 and 11 times, respectively.

#### 2.6.4. Study Limitations and Suggestions

(A) The very limited number of in vivo studies (n = 3) makes their findings non-comparable with the findings of the in vitro studies (n = 75). Hence, the main focus of this review was directed at in vitro studies.

(B) Since our main goal by designing this review was to compare the abilities of different direct and indirect approaches to each other, we only investigated gene expressions that were examined in at least one approach of each of the direct and indirect groups. The expression of BMP1, BMP2, OC, CAP and CEMP1 genes were only examined in Indirect1. Since we did not have any study with a direct approach examining the expression of the mentioned genes, we were unable to include them.

(C) Given the fact that indirect approaches outshone direct ones in most of the categories of outcomes, we suggest that scientists and manufacturers design and use more indirect approaches in vivo. By doing this, we can discover whether the superiority of indirect approaches in vitro also applies for in vivo cases.

(D) There was a significant difference in the number of experiments that each approach was used for, and since the whole purpose of this review was to compare different approaches’ abilities, if the number of experiments was equal, this comparison would have had much more value. We suggest designing a broad and comprehensive in vitro study focusing on all five different approaches and all the different outcomes. By doing this, not only would we have an equal number of experiments in each group, we would also be able to trust the compared results even more due to them being published by one group of authors instead of us comparing the results of different studies conducted by different groups of authors.

(E) Indirect pulp capping (IPC) is one of the few indirect approaches in VPT that is used commonly in clinics. However, IPC requires at least 0.5 mm of residual healthy dentine left on top of the pulp. Therefore, in cases of non-existing dentine, direct pulp capping (DPC) is performed. The results of this review suggest that indirect approaches lead to much better outcomes in almost all categories of results in vitro. Given the superiority of IPC to DPC in in vitro studies, we suggest that more scientists lean onto using and inventing different hydrogels and other biomaterials to simulate IPC when there is not enough healthy dentine left on top of the pulp to perform a conventional IPC. The biomaterials used to simulate IPC must have selective penetration and permeability, just like healthy dentine. This way, hDPSCs would not be in direct contact with hCSCs, and the integrity of the pulp would also be preserved.

(F) In this review, we did not investigate the outcome differences of different cellular assays, cellular culture conditions, and different resource variations in our included studies, for the following reasons: A) for a proper comparison amongst different cellular assays (e.g., MTT, XTT, LDA, SEM, MTS, CCK-8, etc.), we had to compare studies that had complete similarity in all other elements of their study (e.g., similar type of stem cells, similar type of hCSCs, similar setting time, similar setting condition, etc.), and this level of similarity was not available in our included studies; B) Even if we found enough similar studies to compare their outcomes of these cellular assays and cellular culture conditions, classification of these culture conditions and cellular assays would be impossible. For viability/proliferation evaluations, our included studies assessed 19 different assays (MTT, XTT, LDA, SEM, MTS, CCK-8, etc.), and numerous assays were assessed for other evaluations as well, such as cellular attachment (10 assays), cellular migration (4 assays), etc.

## 3. Conclusions

When they are assessed for viability/proliferation and the odonto-/osteogenesis of stem cells in vitro, BD and PRMTA have similarities and both have significantly better outcomes than TCLC, PC and many other commercially available hCSCs in both direct and indirect approaches. Allowing hCSCs to set for at least 24 h in incubation (II) before application results into the most desirable outcomes. Indirect contact between hCSCs and stem cells is significantly less cytotoxic for stem cells and induces remarkably higher rates of odonto-/osteogenesis compared to direct contact in vitro. Moreover, Indirect1 is the most tested contact between hCSCs and stem cells for both viability/proliferation and odonto-/osteogenesis outcomes.

## 4. Materials and Methods

This study has been prepared and organized according to the preferred reporting items for systematic reviews and meta-analyses (PRISMA) 2020 guidelines [42]. This systematic review has been registered at PROSPERO (Registration ID: CRD42023387828). The study question according to the PICO format was as follows: Comparison of biological behavior (O) of stem cells (P) exposed to hCSCs through direct and indirect methods (I) with untreated stem cells (C). Additionally, the stem cells’ behavior with direct exposure was also compared to the hCSCs with indirect exposure.

### 4.1. Eligibility Criteria

#### 4.1.1. Types of Studies

No limitation was considered for the type of the included studies, and all of the in vitro and in vivo articles evaluating the behavior of stem cells that were exposed to cements were included.

#### 4.1.2. Population

All of the studies that used stem cells to analyze the biological features of at least one type of commercially available hCSC through direct or indirect methods were included. We did not apply any restrictions on the type of stem cells (human or animal).

#### 4.1.3. Intervention

Studies that analyzed any type and form of direct and/or indirect contact between hCSCs and stem cells in vitro and in vivo were included.

#### 4.1.4. Control

Studies that considered untreated stem cells as a negative control (NC) group were included.

#### 4.1.5. Types of Outcome Measures

Studies that analyzed the following outcomes were included: (1) Setting time and setting environment of each cement; (2) The types of tests assessed for each type of outcome; (3) Biocompatibility; cellular migration, cellular attachment, cellular viability/proliferation; (4) Odonto-/osteogenesis; ALP activity, mineralization activity (tested via ARS) and odonto-/osteogenesis-related gene expressions.

### 4.2. Information Sources and Search Strategy

An electronic search was executed in Scopus, Google Scholar, and Medline via PubMed to identify eligible studies only in the English language. The search included articles up to 6 December 2022. The search queries mentioned in Table 1 were considered for electronic search.

### 4.3. Study Selection and Data Collection

Two reviewers (AY and SM) independently screened the titles and abstracts of articles and excluded articles based on the exclusion criteria mentioned above. Selected articles were then fully read to see if they passed our inclusion criteria. In the case of any disagreement, a third reviewer (HN) was consulted. The data and outcomes from selected studies were then extracted and tabulated. The same reviewers performed the data extraction, and any conflicts were solved by a third expert (HN).

### 4.4. Data Items

The collected items were as follows: (1) author names, (2) year of publication, (3) type of stem cells, (4) hCSCs and other cements, putties and sealers, (5) additives used to enhance the cements’ abilities, (6) the setting time of each cement, (7) the atmosphere cements were set in (room temperature (RT), in incubator (II), heat dried (HD) and vacuum dried (VD)), (8) control negative group (NC), (9) control positive group, (10) interventions (direct/indirect), (11) assays and tests, (12) evaluation periods, (13) cellular attachment outcomes, (14) cellular migration outcomes, (15) ALP activity, (16) mineralization (ARS), and (17) odonto-/osteogenesis gene expressions.

In reporting the results of gene expression, the following point was considered: For the genes that were expressed in the early phases (e.g., ALP, OCN, OPN, DSPP, and DMP1), only results related to the early phase of the differentiation were mentioned and the comparison related to the late phases of the differentiation for these genes was not mentioned. Similarly, for the late-phase genes (e.g., BMP, OC, and Runx2), merely the comparisons related to the expression in the late phases were noted.

### 4.5. Synthesis Methods

Based on the extracted data, different direct and indirect techniques used for VPT and endodontic treatments were widely diversified. Hence, it was not possible to perform a meta-analysis. A descriptive analysis of the data extracted from clinical studies, along with narrative and graphical synthesis, was performed.

### 4.6. Risk of Bias Assessment

The risk of bias of the included studies was assessed individually by two reviewers (AY and SM), using the CRIS guidelines (checklist for reporting in vitro studies) for in vitro studies and Cochrane’s risk of bias assessment tool for in vivo studies. The CRIS checklist consists of 10 questions, 2 of which were not considered for this review due to not agreeing with the risk of bias analysis of in vitro studies.

## Figures and Tables

**Table 1:** Search queries.

Data Base	Date	Search Query	Result
PubMed	December 2022	(“Dental Pulp stem cells” OR “Stem Cells”[Mesh]) AND (“Dental Cements”[Mesh] OR “mineral trioxide aggregate” [Supplementary Concept] OR “calcium-enriched mixture cement” [Supplementary Concept] OR “tricalcium silicate” [Supplementary Concept] OR “TheraCal” [Supplementary Concept] OR “iRoot BP Plus” [Supplementary Concept] OR “MTA Angelus” [Supplementary Concept] OR “MTA bio” [Supplementary Concept] OR “biodentine” OR “CEM” OR “MTA Plus” OR “MTA Fillapex” OR “endocem” [Supplementary Concept] OR “Neo MTA Plus” OR “MTA Repair HP” OR “Retro MTA” OR “Nex-cem MTA” OR “iRoot SP” [Supplementary Concept] OR “iRoot fast set” OR “well root ST” OR “AH Plus jet” OR “Portland cement” OR “accelerated Portland cement” [Supplementary Concept] OR “bioaggregate” [Supplementary Concept] OR “diaRoot bioaggregate” [Supplementary Concept] OR “NeoPutty” OR “es putty” OR “ERRM” OR “endosequence root repair material” [Supplementary Concept] OR “endosequence BC RRM putty”).	311
Scopus	December 2022	TITLE-ABS-KEY (calcium silicate cement OR calcium silicate-based cement OR MTA OR Biodentine OR TheraCal OR Bioaggregate OR iRoot OR Portland cement OR well root OR AH Plus OR ERRM OR Neoputty OR ES Putty) AND (stem cells OR dental pulp stem cells)	25
Google Scholar	December 2022	(calcium silicate cement OR calcium silicate-based cement OR MTA OR Biodentine OR TheraCal OR Bioaggregate OR iRoot OR Portland cement OR well root OR AH Plus OR ERRM OR Neoputty OR ES Putty) AND (stem cells OR dental pulp stem cells)	347

**Table 4:** Different types of direct and indirect interventions assessed for in vitro and in vivo studies.

Intervention	In Vitro	In Vivo	Description	Number of Articles Featuring the Approach
Direct1	*		Freshly mixed or fully set cements placed at the bottom of plates and cells placed directly on top of them	17 [41,43,46,52,57,59,61,62,63,65,68,69,70,72,78,111,114]
Direct2	*		Cells placed at the bottom of the plates and freshly mixed or fully set cements placed directly on top of them	3 [54,92,98]
Direct3		*	Direct pulp capping procedure in vivo; exposed pulp of teeth directly exposed to hCSCs	1 [45]
Direct4		*	Root end filling procedure in vivo	2 [85,95]
Indirect1	*		Fully set cements placed at the bottom of plates and then incubated with medium, then exposing cells to the supernatant of cements via the diluted medium	24 [41,47,48,51,53,64,66,67,77,79,83,84,88,89,90,91,92,93,94,96,100,102,103,106,107]
Indirect2	*		Transwell plates containing cements placed above the cells and the gap between Transwell plates and cells filled with medium while the medium fully covers the cements.	12 [36,49,50,58,71,79,82,86,101,105,110,115]
Indirect3	*		Freshly mixed cements immediately put in oven to completely dry and then ground into powder and mixed with medium. Filtering the medium and making different dilutions. Exposing cells to dilutions of the medium.	16 [44,55,56,60,73,74,75,76,81,87,97,104,108,112,113,116]
Direct1 + Indirect1	*		Using both Direct1 and Indirect1 approaches simultaneously	3 [38,40,41]
Direct1 + Indirect2	*		Using both Direct1 and Indirect2 approaches simultaneously	1 [39]
Indirect1 + Indirect2	*		Using both Indirect1 and Indirect2 approaches simultaneously	1 [37]

*: Indicating the type of study (i.e., in vitro, or in vivo).

**Table 5:** Comparison of all five different contacts based on their rates of significantly better (SH) results compared to the negative control (NC) group, detailed for each of the outcomes in vitro.

Different Experimented Abilities	>80% SH	50–80% SH	33–50% SH	<33% SH
Cellular attachment	Indirect3	Indirect1	-	Direct1
Viability/proliferation	-	Indirect1	Indirect2 > Indirect3 > Direct1	Direct2
Cellular migration	-	Direct1 > Indirect3	Indirect1 > Indirect2	-
ALP activity	Indirect2 = Indirect3 > Indirect1	Direct1	-	-
Mineralization (ARS)	Direct2 = Indirect2 = Indirect3	Indirect1 > Direct1	-	-
ALP gene expression	Indirect2	Indirect1 > Indirect3	-	Direct1
Runx2 gene expression	Direct2 > Indirect1 ≅ Indirect2	Indirect3	Direct1	-
DSPP gene expression	Indirect3 > Indirect1 > Indirect2	Direct1	-	-
DMP1 gene expression	Indirect1 = Indirect2 = Indirect3	-	Direct1	-
OCN gene expression	Indirect3 > Indirect1 > Indirect2	Direct1	-	-
COL1 and COL1A1 gene expression	Direct1 = Indirect2 = Indirect3	Indirect1	-	-
BSP gene expression	Indirect1 = Indirect2 = Indirect3	-	-	-
OPN gene expression	Indirect3	-	-	Direct1
ON gene expression	Indirect2	Indirect1	-	-

**Abbreviations:** alkaline phosphatase (ALP), alizarin red staining (ARS), runt-related transcription factor 2 (Runx2), dentin sialophosphoprotein (DSPP), dentin matrix acidic phosphoprotein 1 (DMP1), osteocalcin (OCN), collagen type 1 (COL1), bone sialoprotein (BSP), osteopontin (OPN) and osteonectin (ON).

## Data Availability

Non-applicable.

## Supplementary Materials

The following supporting information can be downloaded at: https://www.mdpi.com/article/10.3390/jfb14090446/s1, Figure S1: Risk of bias assessment for in vitro studies; Figure S2: Risk of bias assessment for in vivo studies; Table S1: Complete list of all of the abbreviations in alphabetic order; Table S2: Commercially available hCSCs used in in vitro and in vivo studies; Table S3: Modified hCSCs used in in vitro and in vivo studies.
