# Supplementary material for: Comparative Biocompatibility and Odonto-/Osteogenesis Effects of Hydraulic Calcium Silicate-Based Cements in Simulated Direct and Indirect Approaches for Regenerative Endodontic Treatments: A Systematic Review
Source: J Funct Biomater. 2023 Aug 29;14(9):446. doi: 10.3390/jfb14090446 (PMC10532331; doi:10.3390/jfb14090446)
Supplement: Supplementary file 1 [file jfb-14-00446-s001.zip › jfb-2226630-supplementary.pdf]

Table S1. Complete list of all of the abbreviations in alphabetic order.

Number	Abbreviated Form	Full Form
1	ALP	Alkaline phosphatase
2	ARS	Alizarin red staining
3	BA	Bioaggregate
4	BD	Biodentine
5	BrdU	Bromodeoxyuridine
6	BSP	Bone Sialoprotein
7	CAA	Cellular attachment assay
8	CCK-8	Cell counting kit-8
9	CEM	Calcium enriched matrix
10	CH	Calcium hydroxide
11	CLSM	Confocal laser scanning microscopy
12	COL1	Collagen Type I
13	COP	Copaifera reticulata
14	CP	Control positive
15	DH	Dried heat
16	DMP1	Dentin Matrix Acidic Phosphoprotein 1
17	DPC	Direct pulp capping
18	DSPP	Dentin Sialophosphoprotein
19	EG	Emdogain
20	ELISA	Enzyme-linked immunosorbent assay
21	ERRM	Endo sequence putty
22	ERRM-FS	Endo sequence putty fast set
23	FCM	Flow cytometry
24	FM	Freshly mixed
25	GIC	Glass ionomer cement
26	hBMSCs	Human bone marrow stem cells
27	hCSCs	Hydraulic calcium silicate-based cements
28	hDPSCs	Human dental pulp stem cells
29	hPDLSCs	Human periodontal ligament stem cells
30	hTGSCs	Human tooth germ stem cells
31	hUVESCs	Human umbilical vein endothelial cells
32	IF	Immunofluorescence assay
33	II	In incubation
34	IPC	Indirect pulp capping
35	iRBP	iRoot BP
36	iRFS	iRoot fast set
37	IRM	Intermediate Restorative Material (ZOE)
38	iRSP	iRoot SP
39	LDA	live-dead assay
40	MTA	Mineral trioxide aggregate
41	MTT	3-(4,5-dimethylthiazol- 2-yl)-2,5-diphenyltetrazolium bromide) tetrazolium

42	NC	Negative control
43	NM	Not mentioned
44	NSD	No significant difference
45	OCN	Osteocalcin
46	ON	Osteonectin
47	OPN	Osteopontin
48	PC	Portland cement
49	PCM	Phase contrast microscopy
50	PRMTA	ProRoot MTA
51	PRISMA	Preferred reporting items for systematic reviews and meta-analyses
52	rBMSCs	Rat bone marrow stem cells
53	rDPSCs	Rat dental pulp stem cells
54	RET	Regenerative endodontic treatments
55	RT	Room temperature
56	RT-PCR	Real time - polymerase chain reaction
57	Runx2	Runt-Related Transcription Factor 2
58	SCAP	Stem cells from apical papilla
59	SEM	Scanning electron microscopy
60	SH	Significantly higher
61	SHED	Stem cells from human exfoliated deciduous teeth
62	SL	Significantly lower
63	SRB	Sulforhodamine B colorimetric assay
64	TC	TheraCal
65	TCLC	TheraCal LC
66	TCPT	TheraCal PT
67	TF	TotalFill
68	TMA	Transwell^{TM} migration assay
69	VPT	Vital pulp therapy
70	WB	Western blot
71	WHA	Wound healing assay
72	WRST	Well root ST
73	WST-1	Water-soluble tetrazolium salt
74	XTT	2,3-bis-(2-methoxy-4-nitro-5-sulfophenyl)-2H-tetrazo- lium-5-carboxanilide

Table S2. Commercially available hCSCs used in in vitro and in vivo studies.

Number	Commercial name of the cements	Producer(s) (Country)	Number of appearances in included articles
1	ProRoot MTA	Dentsply Tulsa Dental (USA)	40[35, 37, 39, 40, 42, 44-48, 50, 52-59, 62-68, 71, 72, 74-78, 81, 82, 84, 85, 87, 89]
2	Biodentine	1) Septodont (USA), 2) Septodont (France), 3) Dentsply (Germany)	34[33, 34, 37, 39, 41, 43-45, 47, 49-51, 53, 56, 60, 61, 66-70, 73, 74, 76, 78-80, 82, 83, 85, 86, 88-90]
3	MTA (the brand of the MTA used, was not mentioned by the authors)	NM	8[73, 105-109, 111, 112]
4	MTA Angelus	Angelus Dental Solutions (Brazil)	8[33, 36, 49, 79-81, 87, 94]
5	MTA Plus	Prevest DenPro (USA)	1[47]
6	MTA Fillapex	Angelus Dental Solutions (Brazil)	6[47, 81, 91, 94, 98, 99]

7	Neo MTA Plus	Avalon Biomed (USA)	3[36, 60, 85]
8	MTA Repair HP	Angelus Dental Solutions (Brazil)	3[36, 60, 84]
9	Retro MTA	BioMTA (Korea)	1[67]
10	Nex-cem MTA	GC (Japan)	1[43]
11	Endoseal MTA	Maruchi (Korea)	1[97]
12	OrthoMTA	BioMTA (Korea)	1[90]
13	iRoot BP Plus	Innovative Bioceramix (Canada)	1[40]
14	iRoot SP	Innovative Bioceramix (Canada)	2[91, 101]
15	iRoot fast set (iRoot FS)	1) Innovative Bioceramix (Canada), 2) Dentsply Tulsa Dental (USA)	3[61, 77, 83]
16	Well Root ST	Dentsply (Germany)	1[45]
17	AH Plus	Dentsply (Germany)	4[91, 94, 98, 99]
18	CEM	Bionique Dent (Iran)	5[36, 80, 81, 87, 90]
19	TheraCal LC	1) Bisco (USA), 2) Bisco (France)	9[33, 37, 41, 49, 78, 80, 82, 86, 89]
20	TheraCal PT	Bisco (France)	1[86]
21	Portland Cement	1) Mapei p.a. (Italy), 2) Italcementi Group (Italy)	4[48, 59, 64, 92, 96]
22	Bioaggregate	Innovative Bioceramix (Canada)	1[73]
23	NeoPutty	NuSmile (USA)	2[100, 104]
24	ES Putty	Brasseler (USA)	5[68, 74, 97, 100, 104]
25	ES Fast set	Brasseler (USA)	1[74]
26	Ceraseal	Meta Biomed Co. (Korea)	1[97]
27	TotalFill BC	FKG Dentaire (Switzerland)	2[44, 99]
28	BioRoot RCS	Septodont (France)	1[88]

Table S3. Modified hCSCs used in in vitro and in vivo studies.

Number	Commercially available hCSCs with modifications	Number of appearances in articles
1	MTA + COP (Copaifera reticulata)	1[105]
2	ProRoot MTA + LPS	1[95]
3	RetroMTA + LPS	1[95]
4	BD + LPS	1[95]
5	PC + ZnO	1[96]

6	PC + ZrO_2	1[96]
7	ProRoot MTA + 2% FBS	1[46]
8	Ceria – incorporated MTA	1[106]
9	MTA + MM (mineralization medium)	2[76, 111]
10	MTA + PRP	1[57]
11	ProRoot MTA + DW (distilled water)	1[57]
12	ProRoot MTA + 2.5% Na_2HPO_4	1[58]
13	ProRoot MTA + 5% $CaCl_2$	2[46, 58]
14	MTA + EMD (enamel matrix deprived)	1[112]
15	White ProRoot MTA – enriched PPC (polymeric powder coatings) (WMPPC)	1[110]
16	Gray ProRoot MTA – enriched PPC (polymeric powder coatings) (GMPPC)	1[110]
17	Accelerated – set white ProRoot MTA (AWMTA)	1[103]
18	Accelerated – set malaysian white PC (AMWPC)	1[103]

Author (Year)	Joanne Briggs Institute (JBI)							
	(1)	(2)	(3)	(4)	(5)	(6)	(7)	(8)
All 75 in vitro studies	?	?	?	+	+	+	+	+

JBI – Critical appraisal checklist for in vitro studies

(1) Was the assignment to treatment groups truly random?
(2) Was allocation to treatment groups concealed from the allocator?
(3) Were those assessing the outcomes blind to the treatment allocation?
(4) Were control and treatment groups comparable at entry?
(5) Were groups treated identically other than for the named interventions?
(6) Were outcomes measured in the same way for all groups?
(7) Were outcomes measured in a reliable way?
(8) Was appropriate statistical analysis used?

Legend	
+	Yes
–	No
?	Unclear

Figure S1. Risk of bias assessment for in vitro studies.

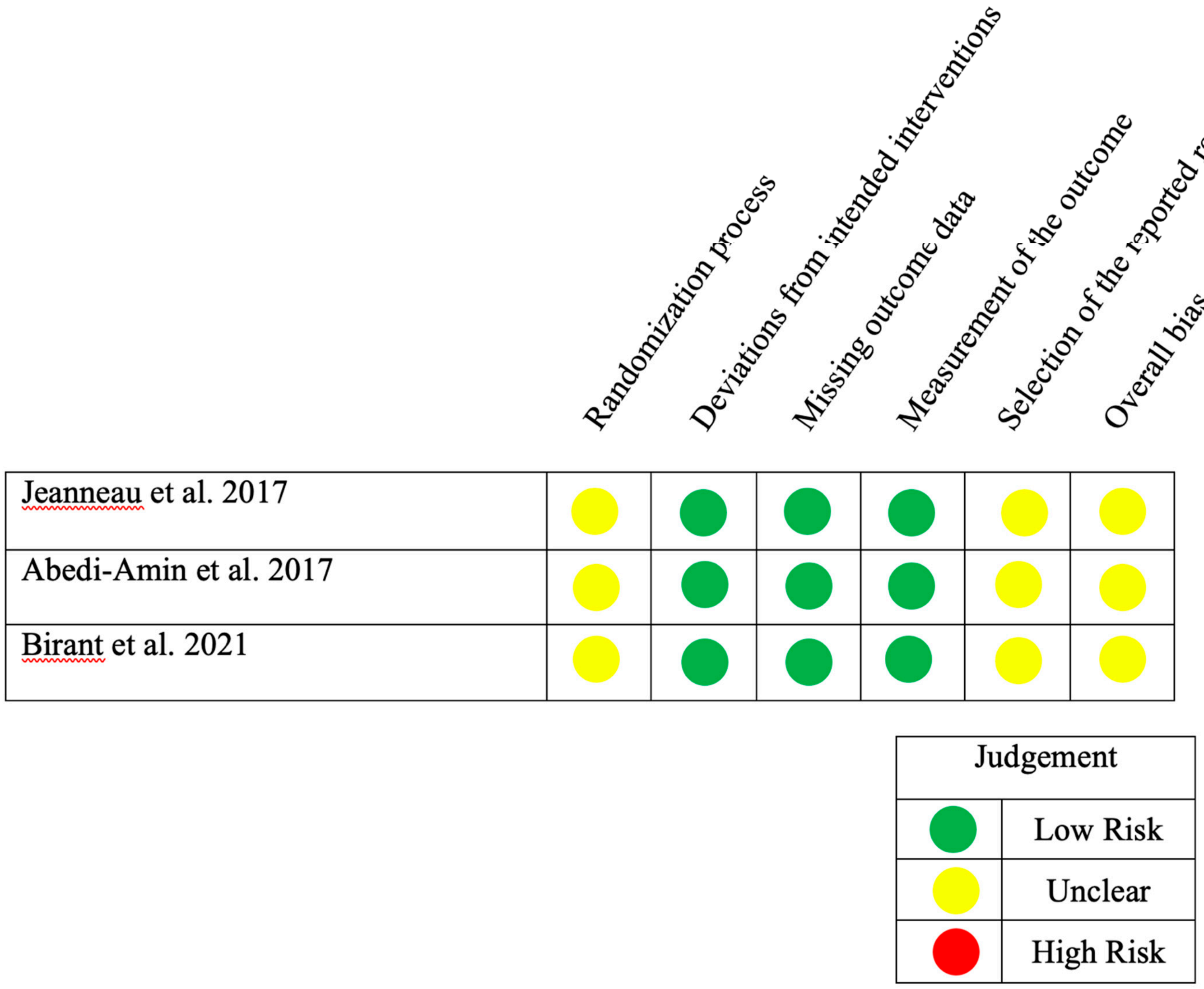

Figure S2. Risk of bias assessment for in vivo studies.
